# Effects of the Otago Exercise Program on Physical Function Performance Related to Falls in the Elderly: A Meta-Analysis of Randomized Controlled Trials

**DOI:** 10.3390/healthcare14142187

**Published:** 2026-07-20

**Authors:** Yingxue Tang, Jing Shi, Zhaofeng Meng, Yong Zhang

**Affiliations:** 1 School of Medicine, Shaoxing University, Shaoxing 312000, China; 24021054118@usx.edu.cn (Y.T.); 24021054115@usx.edu.cn (J.S.); 2 College of Physical Education and Health Sciences, Zhejiang Normal University, Jinhua 321004, China

**Keywords:** otago exercise program, fall, physical function, meta-analyses

## Abstract

**Highlights:**

**What are the main findings?**

- In OEP interventions targeting the risk of falls among older adults, group interventions appear to yield greater improvements than home-based interventions.
- In OEP interventions targeting the risk of falls among older adults, interventions lasting four months or longer may help achieve more sustained benefits.

**What are the implications of the main findings?**

- Group-based OEP interventions lasting at least four months may be associated with greater improvements in physical function related to fall risk in older adults.

**Abstract:**

**Background/Objectives**: This meta-analysis primarily aimed to evaluate the effects of the Otago Exercise Program (OEP) on physical function performance related to the risk of falls in the elderly. **Methods**: A literature search was conducted in PubMed, Embase, Cochrane Library, and Web of Science for studies on OEP interventions related to falls in older adults. Eligible studies were randomized controlled trials that reported at least one physical function outcome related to fall risk, including the Timed Up and Go test (TUG), Berg Balance Scale (BBS), Short Physical Performance Battery (SPPB), 30-Second Sit-to-Stand Test (30s-SST), Falls Efficacy Scale-International (FES-I), or 6-Minute Walk Test (6MWT). Subsequently, a risk of bias assessment, as well as data extraction and synthesis, was performed. **Results**: Data were synthesized from a total of 13 studies, involving 1278 participants from OEP (*n* = 660) and control (*n* = 618) trials. The results suggested that OEP was associated with improvements in TUG (WMD = −1.47; 95%CI: −2.44, −0.51), BBS score (WMD = 4.43; 95%CI: 2.50, 6.36), SPPB scores (WMD = 0.76; 95%CI: 0.43, 1.09), 30s-SST (WMD = 2.68; 95%CI: 0.86, 4.49), FES-I score (WMD = −1.74; 95%CI: −3.15, −0.33). However, no significant effect was observed for the 6MWT. Subgroup analysis suggested that group-based OEP appeared to provide greater improvements in TUG, BBS scores, and 30s-SST, whereas home-based OEP did not show significant effects. In addition, interventions lasting ≥4 months were associated with greater improvements for TUG, BBS scores, and the 30s-SST. Older adults aged ≥75 showed significant improvements in TUG and 30s-SST. However, substantial heterogeneity was observed across several outcomes, and evidence for SPPB, FES-I, and 6MWT was based on a limited number of studies. **Conclusions**: OEP may improve physical function performance related to fall risk in older adults, such as the TUG, BBS, SPPB, 30s-SST, and FES-I, while no significant effect was observed for the 6MWT. Importantly, the findings support improvements in physical function performance rather than a direct reduction in actual fall incidence. These findings should be interpreted cautiously due to substantial heterogeneity and the limited number of trials for some outcomes. A group-based OEP lasting at least 4 months may provide greater benefits for improving physical function related to fall risk, particularly among adults aged ≥75 years.

## 1. Introduction

A fall refers to an unexpected body descent caused by a loss of balance or an unstable center of gravity [1], representing a significant threat to elderly health. Among individuals aged 65 and older, approximately 30% experience falls annually [2]. A U.S. survey study [3] revealed that falls account for over 90% of unintentional injuries among older adults. Falls not only cause acute injuries [4], but also exert persistent and severe impacts on the physical functioning of the elderly [5]. Research has confirmed that older adults who experience falls subsequently exhibit a marked decline in activities of daily living capabilities, reduced physical activity, impaired self-care abilities, and a persistent trend of functional deterioration [6,7]. Therefore, fall prevention is a critical objective in the daily health management of older adults.

Falls among older adults are closely associated with impairments in multiple physical functions [8,9,10]. From a functional perspective, the risk of falling is not determined by a single physical impairment but rather by the interaction between balance control, lower-limb strength, gait adaptability, endurance, and psychological factors [11]. Impaired balance makes it more difficult for older people to maintain physical stability and reduces their ability to cope with external disturbances [12], whilst a decline in lower-limb muscle strength limits their ability to move around and climb stairs [13]. Gait disturbances and reduced endurance may further impair older people’s ability to walk safely in their daily lives, particularly when they are feeling tired [14,15,16]. Furthermore, the fear of falling may lead to restricted activity and a decline in physical function, thereby increasing the risk of future falls [17]. As a key non-medical intervention to address these risk factors, exercise is widely employed in fall prevention among older adults. However, the long-term efficacy of isolated lower-limb strength training or balance training in reducing falls remains uncertain when considering the multifaceted physical demands of community living activities [18,19,20]. Research has confirmed that multidimensional exercise programs incorporating strength, balance, gait, and aerobic components yield more significant reductions in fall frequency compared to single-type exercise [21,22,23].

The Otago Exercise Program (OEP) is a home-based exercise program specifically designed for older adults to prevent falls. The benefits of this program in preventing falls and improving physical function become more pronounced as older adults age [24]. It had also been applied to fall prevention in stroke patients [25] and to enhance physical function in elderly individuals with sarcopenia [26]. OEP primarily incorporates strength, balance, and gait training. It employs a progressive training approach to gradually enhance older adults’ balance control, thereby reducing falls [27]. Research has demonstrated that OEP, utilizing a combined exercise program, holds distinct advantages over single-function training in preventing home falls [28]. However, in real-world communities and social settings, beyond lower-body strength and balance function, cardiopulmonary fitness, muscular endurance, and cognitive function also play crucial roles in sustaining prolonged physical activity and maintaining postural control. Therefore, comprehensively analyzing the effects of OEP on physical function performance related to fall risk in older adults is particularly important. Currently, the Timed Up and Go test (TUG), Berg Balance Scale (BBS), Short Physical Performance Battery (SPPB), 30-Second Sit-to-Stand Test (30s-SST), Falls Efficacy Scale-International (FES-I), and 6-Minute Walk Test (6MWT) are commonly used to assess functional performance related to falls. These physical function performances support the stability and endurance of physical transport in older adults.

Although previous systematic reviews and meta-analyses have examined the relationship between OEP and the incidence of falls and balance ability, there are still limitations. Most studies have focused primarily on the incidence of falls, whilst research on physical function related to the risk of falls is relatively limited. Furthermore, it is also unclear whether the effects of OEP differ by age, intervention modality, duration, or frequency. In particular, although OEP was originally designed as a home-based exercise program for older adults in the community [29], it is now also delivered in group-based or hybrid intervention formats; however, the impact of different intervention models on physical function outcomes remains to be further clarified.

In terms of intervention models, the OEP can be undertaken independently by participants in their own homes, or delivered through group sessions or a blended approach. Compared with a home-based program, group-based OEP may offer further advantages. Supervision by professionals ensures exercise safety and movement quality, whilst real-time corrections help participants perform the exercises more accurately. Group-based OEP may also improve adherence through fixed participation times, social support, and peer encouragement [30,31]. In contrast, whilst home-based OEP offers greater flexibility and accessibility, its efficacy is often limited by a lack of supervision, low adherence, and insufficient exercise intensity or quality. The differences in outcomes between these two distinct intervention models, as well as variations in intervention duration and frequency, also need to be clarified.

The effectiveness of OEP interventions may also be related to the age of older adults [24,32]. Significant heterogeneity exists in the physical functional status of older adults across different age groups [33,34,35]. Previous studies indicate that older adults experience greater impairment in balance, lower-limb muscle strength, and movement and fall control with increasing age [36,37,38]. The incidence of falls increases with age, with individuals aged 75 and older experiencing approximately twice the fall rate as the overall elderly population [4]. Therefore, comparing the effectiveness of OEP interventions across different age groups will help to determine whether they are more suitable for specific high-risk populations.

Based on the above background and perspectives, this paper conducted a meta-analysis of randomized controlled trials (RCTs). Unlike previous meta-analyses that mainly focused on fall incidence or the overall effects of OEP, this study examined the effects of OEP on physical function performance related to fall risk in older adults. It further explored whether these effects differed across age groups and intervention models. The aim was to provide useful evidence for understanding how OEP may improve physical function related to fall risk in older adults and to inform future fall-risk-reduction strategies.

## 2. Materials and Methods

We conducted the systematic review and meta-analysis following the Preferred Reporting Items for Systematic Reviews and Meta-Analyses (PRISMA) guidelines [39]. The systematic review was registered with PROSPERO (CRD420251102323).

### 2.1. Data Sources and Search Strategy

Literature searches were conducted using electronic databases including PubMed, Embase, Cochrane Library, and Web of Science. Articles published up to 22 January 2026 were searched using a combination of subject and free-text terms in the title and abstract. These terms included “Otago exercise program”, “Otago exercise”, “OEP”, “Otago”, “falling”, “fall”, “accidental fall”, and “fall and slip”. The complete search strategies for all databases are provided in the Supplementary Materials.

### 2.2. Literature Inclusion and Exclusion Criteria

Two authors (Yingxue Tang and Jing Shi) independently conducted the literature search, screening, data extraction, and quality assessment. Another author (Zhaofeng Meng) provided additional review and insights. Any disagreements regarding article inclusion or exclusion were discussed and resolved by all authors. To ensure the comprehensiveness of the search, no restrictions were imposed on the year of publication during the search phase, and potentially relevant gray literature records were not excluded during the initial screening process.

**Figure 1:**
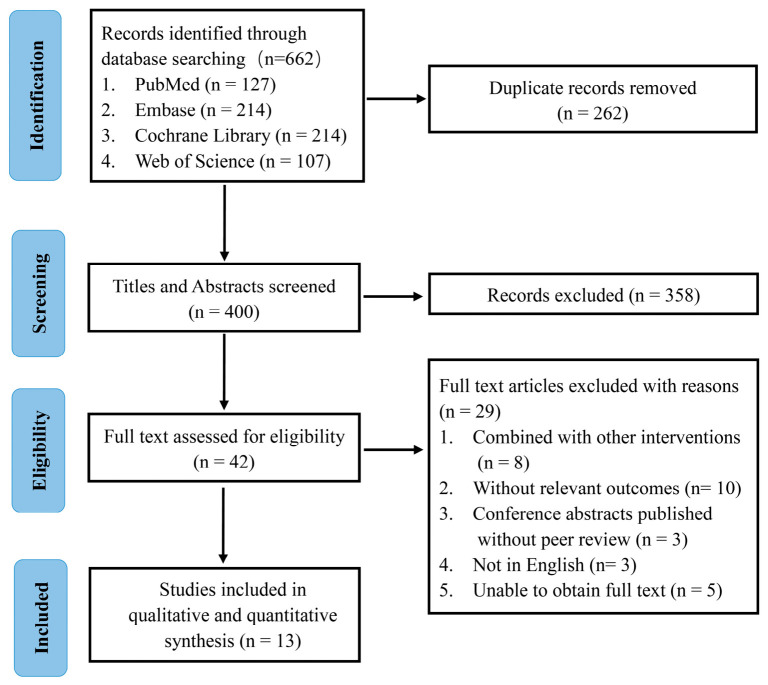
Literature Screening and Study Inclusion Flowchart.

Initially, the titles and abstracts of identified articles were screened for relevance, followed by retrieval and review of the full texts of selected articles. The following criteria were used to include articles in this review: (1) studies were RCTs and published in English; (2) participants aged 65 years or older; (3) the experimental group received only OEP as an intervention, while the control group received other interventions; (4) study outcomes must include at least one of the following: TUG, BBS, SPPB, 30s-SST, FES-I, or 6MWT. Exclusion criteria were as follows: (1) non-RCTs or those without a control group; (2) non-OEP or those combined with other interventions; (3) studies lacking fall-related physical function performance data; (4) conference abstracts that are not peer-reviewed; (5) non-English publications. The detailed process for article screening and inclusion is shown in Figure 1. In addition, it should be noted that the relevant gray literature was not excluded at the search stage. However, the final analysis only included RCTs that were published in English, reviewed by peers, available as full texts, and had data that could be extracted. Consequently, during the screening of the full-text articles, we excluded papers that had not undergone peer review, conference abstracts, and other forms of gray literature. This exclusion may have introduced publication and language bias.

### 2.3. Bias Risk Assessment

In this study, we used the Cochrane Collaboration tool to assess the risk of bias. The initial assessment was performed by two authors (Yingxue Tang and Jing Shi), and any disagreements were rectified by discussing them with other authors (Zhaofeng Meng and Yong Zhang). The source of bias, including selection (random sequence generation and allocation concealment), performance (blinding of participants and personnel from the OEP intervention), detection (blinding of outcome assessment), attrition (incomplete outcome data), reporting (selective reporting), and other biases, was detected. In the main meta-analysis, all RCTs meeting the inclusion criteria were included. Studies assessed as having a high risk of bias were not excluded solely because of their risk of bias. Their potential impact on the results was further examined through sensitivity analyses.

### 2.4. Data Extraction

Information and data from 13 eligible articles were independently extracted by two authors (Yingxue Tang and Jing Shi). The data included study characteristics (authors, publication year), participant demographics (age, gender), OEP variables (intervention duration, frequency, and model), and outcomes (TUG, BBS, SPPB, 30s-SST, FES-I, 6MWT) in Table 1. Outcome values are presented as the mean ± standard deviation (SD).

### 2.5. Outcomes and Subgroups Division

As commonly used indicators for evaluating physical function performance in older adults, data from the TUG, BBS, SPPB, 30s-SST, FES-I, and 6MWT were utilized for meta-analysis. The TUG assesses an individual’s comprehensive functional ability in standing up, walking, and turning. An increased TUG score often indicates elevated fall risk [52]. The BBS evaluates static and dynamic balance capabilities through multiple balance tasks, with a reduced BBS score being highly correlated with fall risk [53]. The SPPB, comprising balance, gait speed, and sit-to-stand tests, comprehensively reflects lower-limb function and physical performance. It is also a significant predictor of fall risk [54]. The 30s-SST assesses functional lower-limb strength, as insufficient functional muscle strength in the lower limbs is a key factor in gait instability and falls [55]. The FES-I measures participants’ level of fall-related anxiety, as fear of falling can lead to reduced activity and further increase fall likelihood [56]. The 6MWT evaluates walking endurance and cardiopulmonary function, with diminished 6MWT performance often accompanying reduced mobility and being associated with fall risk [57]. In this study, we conducted a meta-analysis incorporating the aforementioned indicator data to comprehensively evaluate the effect of OEP on physical function performance related to the risk of falls in the elderly.

Differences in age and implementation models may influence intervention outcomes. Therefore, subgroup analyses were conducted when moderate or substantial heterogeneity was observed [58]. Based on the data characteristics of the included studies, we conducted subgroup analyses according to the following criteria: implementation model (“group-based training and home-based training”), age (<75 years and ≥75 years) [59,60], intervention duration (<4 months and ≥4 months), and intervention frequency (twice weekly and thrice weekly). These may help provide evidence for developing more targeted and adaptive OEP implementation strategies.

Subgroup analyses were conducted in the study based on clinically relevant factors and the distribution of characteristics across the included studies. The age cutoff of 75 years was selected because previous gerontological and clinical studies have commonly classified adults aged 75–84 years as middle-old adults, and the Japan Gerontological Society and the Japan Geriatrics Society have also proposed 75 years as a clinically meaningful threshold for defining older age [59]. In addition, Sherrington et al. [61], in a systematic review conducted to inform the WHO guidelines on physical activity and sedentary behavior, used participant age above 75 years as a subgroup factor when examining the effects of exercise on fall prevention. Intervention duration was categorized as <4 months and ≥4 months because the included trials were mainly distributed between shorter interventions of approximately 2–3 months and longer interventions of 4–6 months. This cutoff allowed comparison between relatively short-term and longer-term OEP implementation while maintaining an appropriate distribution of studies across subgroups. Intervention frequency was categorized as twice weekly and three times weekly because these were the main frequencies reported in the included trials and reflected the common implementation patterns of OEP in the available evidence.

### 2.6. Data Synthesis and Meta-Analysis

Review Manager (RevMan, version 5.4; The Cochrane Collaboration, Copenhagen, Denmark) was employed for the meta-analysis and risk of bias assessment. Stata Statistical Software (Release 16; StataCorp LLC, College Station, TX, USA) was used for the publication bias and sensitivity analyses. The primary statistical procedures in this study were the computation, heterogeneity, and verification of the combined effect size. Effect sizes were reported as weighted mean differences (WMDs) with 95% confidence intervals (CIs). A heterogeneity analysis was conducted on the included studies. The fixed-effects model was selected when I^2^ < 50%, and the random-effects model was selected when I^2^ > 50%. Publication bias was assessed using funnel plots and Egger’s regression test. Data visualization combined forest plots to illustrate effect estimates and corresponding 95%CIs, while sensitivity analyses were conducted to evaluate the robustness of the results. In the sensitivity analyses, the robustness of the pooled results was assessed by sequentially excluding individual studies to determine whether any single study had a significant impact on the overall effect estimate and heterogeneity. In addition, the certainty of evidence for each outcome was assessed using the GRADE approach.

## 3. Results

### 3.1. Characteristics of Included Studies

A total of 662 related articles were identified from the electronic databases, including PubMed, Embase, Cochrane Library, and Web of Science. After excluding 262 duplicate studies, 400 studies remained. Following a review of titles and abstracts, 42 studies were retained after excluding non-randomized controlled trials and irrelevant research. Upon further full-text review, studies failing to meet inclusion criteria were excluded, ultimately resulting in 13 studies being included in the meta-analysis. The literature screening process is illustrated in Figure 1.

The 13 included RCTs encompassed 1278 participants, comprising 660 in the intervention group and 618 in the control group. Among these, 8 studies involved group-based OEP interventions, while 5 studies utilized home-based OEP interventions. The included studies varied in terms of participants’ age, sample size, intervention duration, exercise frequency, intervention mode, and control conditions. Some studies used usual care as the control, whereas others used active control interventions, such as Gaze stability exercises. In several studies, the usual care condition was not described in detail. Regarding outcomes, compared with the control group, the OEP group showed a decreasing trend in TUG and FES-I, and an increasing trend in BBS, SPPB, 30s-SST, and 6MWT. Detailed characteristics are presented in Table 1.

### 3.2. Risk of Bias of the Included Studies

**Figure 2:**
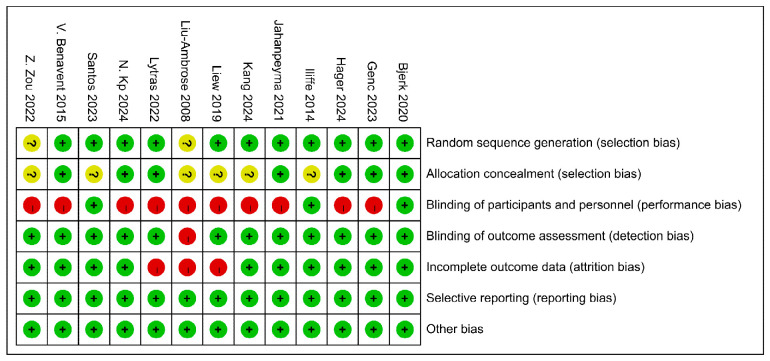
Summary of the risk of bias for the trials included in this meta-analysis. Green, red, and yellow indicate low, high, and unclear risk of bias, respectively; “+”, “−”, and “?” denote low, high, and unclear risk of bias [27,40,41,42,43,44,45,46,47,48,49,50,51].

The detailed judgment of risk of bias derived from the Cochrane Collaboration tool was depicted in Figure 2. The overall methodological quality of the studies included in the review was acceptable. Most studies adequately reported the generation of the random sequence and the concealment of allocation, and the overall risk of selection bias was low. Some trials did not use blinding for either participants or researchers and were therefore considered to be at high risk of performance bias [27,40,42,43,44,46,47,49,50,51]. Although it is difficult to blind participants and researchers in exercise intervention studies, the lack of blinding may still influence the study results. Participant adherence, motivation, and the interaction between researchers and participants may all influence the relevant outcomes. This issue should be considered as an important methodological limitation when interpreting the effect estimates. In addition, a study in which the researchers both implemented the intervention and assessed the outcomes was considered to have a high risk of detection bias [27]. Furthermore, the results section of this study mentions that two outliers were identified and excluded based on the distribution of fall counts, which was considered to indicate a potential risk of attrition bias [27]. The exclusion of outliers may have affected the stability of the original study findings and introduced uncertainty regarding their contribution to the results of this meta-analysis. Two other studies reported significant follow-up loss due to various confounding factors and were therefore considered to have a high risk of attrition bias [47,51]. To examine whether studies with high detection bias or attrition bias influenced the overall results, sensitivity analyses were conducted by excluding these studies where applicable. The results of these sensitivity analyses were reported in the corresponding outcome analyses and were considered when interpreting the robustness of the findings. No studies were judged to have a high risk of reporting bias or bias from other sources.

### 3.3. Effect of OEP on TUG in Older Adults

**Figure 3:**
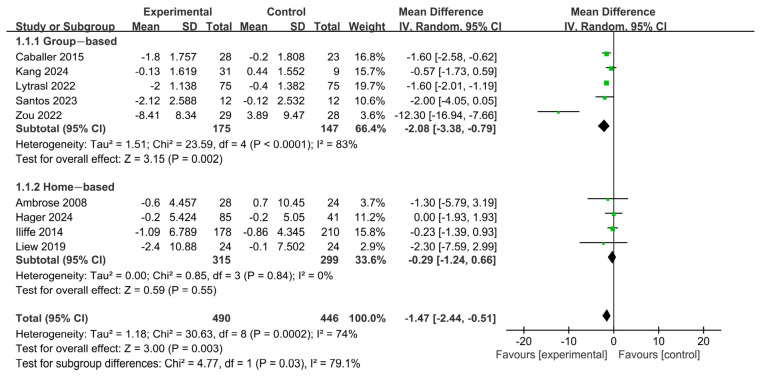
Forest plot of changes in TUG across different intervention models (seconds) [27,40,44,45,47,48,49,50,51].

**Figure 4:**
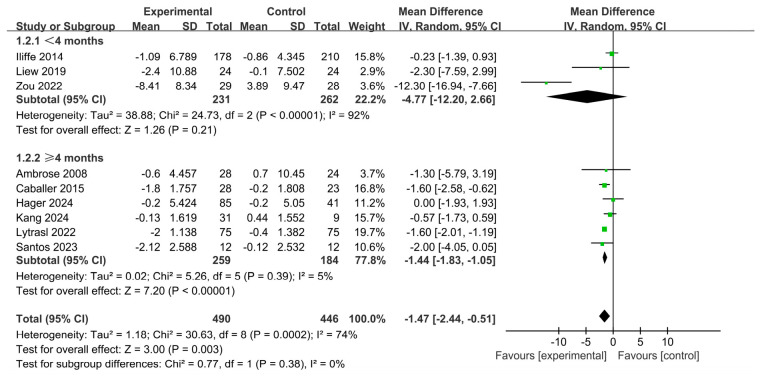
Forest plot of changes in TUG for different intervention durations (seconds) [27,40,44,45,47,48,49,50,51].

**Figure 5:**
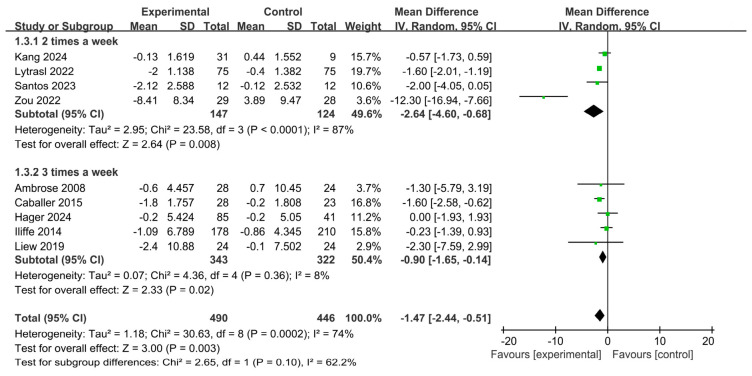
Forest plot of changes in TUG at different intervention frequencies (seconds) [27,40,44,45,47,48,49,50,51].

**Figure 6:**
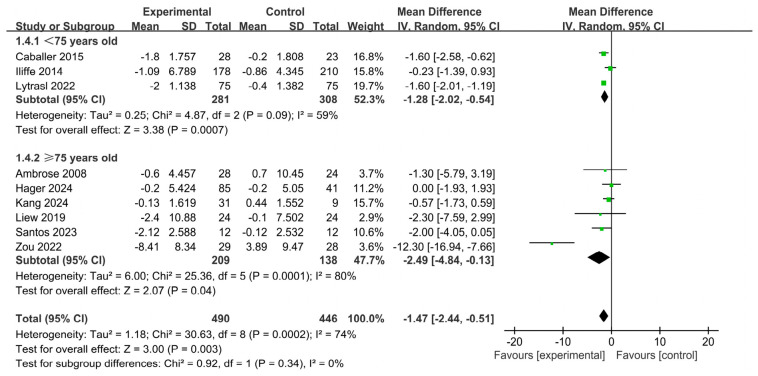
Forest plot of changes in TUG among participants of different age groups (seconds) [27,40,44,45,47,48,49,50,51].

Given the high heterogeneity (I^2^ = 74%, *p* < 0.001), a random-effects model was selected (Figure 3). The results indicated that OEP was associated with a significant improvement in TUG among older adults (WMD = −1.47; 95%CI: −2.44, −0.51; *p* = 0.003). The certainty of evidence was rated as low due to risk of bias and substantial heterogeneity (see Supplementary Materials). Subgroup analysis suggested that group-based interventions were associated with greater improvements in TUG, but substantial heterogeneity remained in this subgroup (WMD = −2.08, 95%CI: −3.38, −0.79, *p* = 0.002; I^2^ = 83%, *p* < 0.001), while home-based interventions showed non-significant effects. Regarding the duration of intervention (Figure 4), interventions lasting ≥4 months yielded significant improvement in TUG (WMD = −1.44; 95%CI: −1.83, −1.05; *p* < 0.001; I^2^ = 5%), whereas interventions lasting <4 months showed non-significant effects. In terms of intervention frequency (Figure 5), twice weekly (WMD = −2.64; 95%CI: −4.60, −0.68; *p* = 0.008) and three times per week (WMD = −0.90; 95%CI: −1.65, −0.14; *p* = 0.02) both demonstrated significant effects. However, there was considerable heterogeneity within the twice-weekly group (I^2^ = 87%, *p* < 0.001). Furthermore, when considering the age of the participants (Figure 6), greater improvement in TUG was observed among adults aged ≥75 years (WMD = −2.49; 95%CI: −4.84, −0.13; *p* = 0.04), although there was substantial heterogeneity (I^2^ = 80%, *p* < 0.001).

Sensitivity analysis was performed using a leave-one-out approach. For the TUG outcome, exclusion of the study by Zou et al. [50] reduced heterogeneity from 74% to 26%, while the combined effect remained statistically significant. This suggests that the study by Zou et al. [50] was a major contributor to heterogeneity in the TUG analysis, whereas the overall direction and statistical significance of the combined effect remained stable. In addition, exclusion of studies with a high risk of bias did not materially change the direction or statistical significance of the pooled effect, although heterogeneity changed to some extent (see Supplementary Materials). Although Egger’s test did not indicate evidence of publication bias, fewer than 10 studies were included in this analysis; therefore, the publication bias assessment should be interpreted with caution due to limited statistical power (see Supplementary Materials).

### 3.4. Effect of OEP on BBS Scores in Older Adults

**Figure 7:**
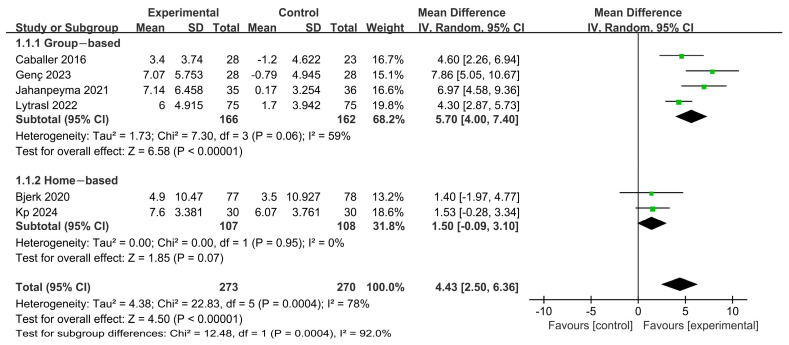
Forest plot of changes in BBS scores across different intervention models [40,41,42,43,46,51].

**Figure 8:**
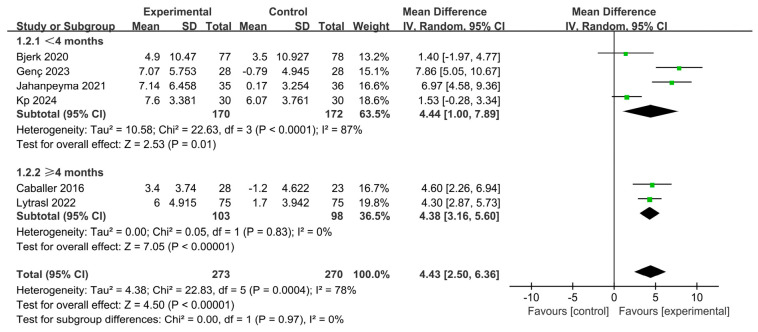
Forest plot of changes in BBS scores for different intervention durations [40,41,42,43,46,51].

**Figure 9:**
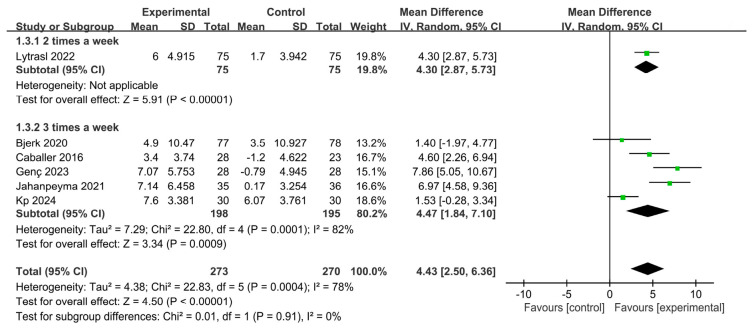
Forest plot of changes in BBS scores at different intervention frequencies [40,41,42,43,46,51].

**Figure 10:**
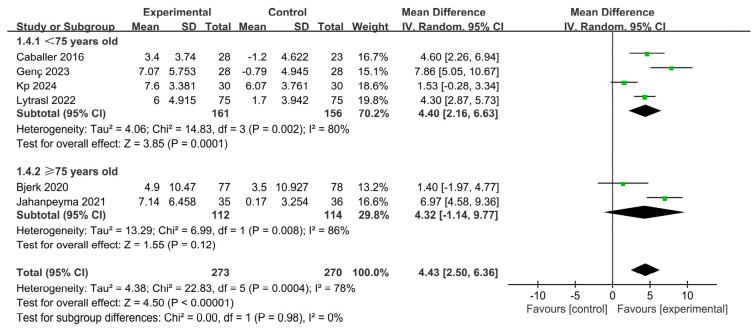
Forest plot of changes in BBS scores among participants of different age groups [40,41,42,43,46,51].

There was substantial heterogeneity among the studies (I^2^ = 78%, *p* < 0.001). Analysis using a random-effects model suggested that OEP significantly improved BBS scores in older adults (WMD = 4.43; 95%CI: 2.50, 6.36; *p* < 0.001). The certainty of evidence was rated as low, mainly due to risk of bias and substantial heterogeneity (see Supplementary Materials). Subgroup analysis revealed that group-based interventions showed significant improvement in BBS scores in older adults (WMD = 5.70; 95%CI: 4.00, 7.40; *p* < 0.001), although there was some heterogeneity (I^2^ = 59%, *p* = 0.06). In contrast, home-based interventions did not produce statistically significant effects (Figure 7). Interventions lasting ≥4 months (WMD = 4.38; 95%CI: 3.16, 5.60; *p* < 0.001) and those lasting <4 months (WMD = 4.44; 95%CI: 1.00, 7.89; *p* = 0.01) both showed positive effects. However, considerable heterogeneity was observed for the shorter interventions (I^2^ = 87%, *p* < 0.001) (Figure 8). A frequency of 3 times per week showed more significant improvements in BBS in older adults but showed high heterogeneity (WMD = 4.47; 95%CI: 1.84, 7.10; *p* < 0.001; I^2^ = 82%, *p* < 0.001) (Figure 9). In addition, a positive change in BBS was observed among older adults aged under 75 (WMD = 4.40; 95%CI: 2.16, 6.63; *p* < 0.001), but there was high heterogeneity (I^2^ = 80%, *p* = 0.002) (Figure 10).

Leave-one-out sensitivity analysis showed that sequentially excluding individual studies had no substantial impact on the direction or statistical significance of the meta-analytic result. However, heterogeneity remained high even after excluding any single study, suggesting that the heterogeneity observed in the BBS analysis was not driven by any single study but may have been due to clinical and methodological differences between the included trials. Sensitivity analysis excluding studies with high risk of bias showed that the pooled effect remained stable and that heterogeneity was not materially reduced. The Egger test results indicated no significant publication bias (see Supplementary Materials). As this analysis included fewer than 10 studies, the assessment of publication bias may be limited.

### 3.5. Effect of OEP on SPPB Scores in Older Adults

**Figure 11:**
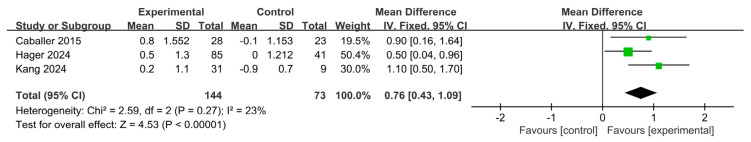
Forest plot of changes in SPPB scores [40,44,49].

The heterogeneity among studies was acceptable (I^2^ = 23%, *p* = 0.27). Analysis using a fixed-effects model suggested a favorable effect of OEP on SPPB scores in older adults (WMD = 0.76; 95%CI: 0.43, 1.09; *p* < 0.001) (Figure 11). The certainty of the evidence was downgraded to low due to a serious risk of bias and imprecision from a limited sample size (see Supplementary Materials). The Egger test results indicated no publication bias (see Supplementary Materials). However, because fewer than 10 studies were included in this analysis, the assessment of publication bias may be limited.

### 3.6. Effect of OEP on 30s-SST in Older Adults

**Figure 12:**
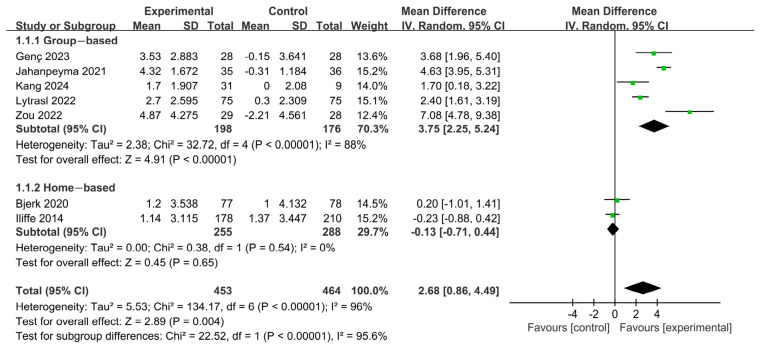
Forest plot of changes in 30s-SST across different intervention models (reps) [41,42,43,45,49,50,51].

**Figure 13:**
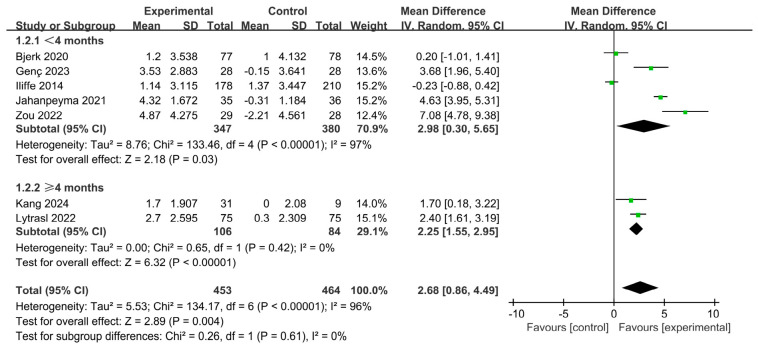
Forest plot of changes in 30s-SST for different intervention durations (reps) [41,42,43,45,49,50,51].

**Figure 14:**
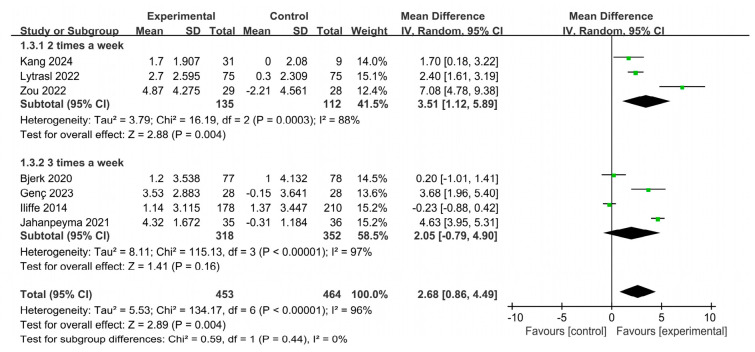
Forest plot of changes in 30s-SST at different intervention frequencies (reps) [41,42,43,45,49,50,51].

**Figure 15:**
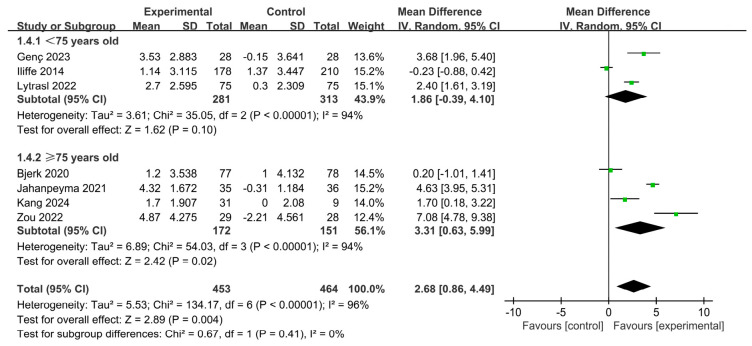
Forest plot of changes in 30s-SST among participants of different age groups (reps) [41,42,43,45,49,50,51].

There was considerable heterogeneity among the studies (I^2^ = 96%, *p* < 0.001). Analysis using a random-effects model suggested that OEP significantly improved 30s-SST in older adults (WMD = 2.68; 95%CI: 0.86, 4.49; *p* = 0.004). The certainty of evidence was rated as low, mainly due to risk of bias and substantial heterogeneity (see Supplementary Materials). Subgroup analysis suggested that group-based interventions can improve 30s-SST performance (WMD = 3.75; 95%CI: 2.25, 5.24; *p* < 0.001), with every study showing positive results. However, the effect of home-based interventions was not statistically significant (Figure 12). In terms of duration (Figure 13), interventions lasting ≥4 months showed more consistent improvement in the 30s-SST (WMD = 2.25; 95%CI = 1.55, 2.95; *p* < 0.001; I^2^ = 0%). Interventions conducted twice weekly showed improvement but exhibited high heterogeneity (WMD = 3.51; 95%CI = 1.12, 5.89; *p* = 0.004; I^2^ = 88%) (Figure 14). In the age-based subgroup analysis, improvement in 30s-SST was observed among individuals aged ≥75 years (WMD = 3.31; 95%CI = 0.63, 5.99; *p* = 0.02), but there was substantial heterogeneity (I^2^ = 94%, *p* < 0.001) (Figure 15).

Leave-one-out sensitivity analysis showed that sequentially excluding individual studies had no substantial impact on the direction or statistical significance of the overall meta-analytic result. However, heterogeneity remained considerable across the leave-one-out analyses, with I^2^ values ranging from 92% to 96%. These findings indicate that the 30s-SST result was stable in terms of effect direction and statistical significance, and that the heterogeneity was not driven by any single study. Overall, the sensitivity analyses showed stable results. Excluding studies with a high risk of bias did not materially change the overall pooled estimate or heterogeneity. Egger’s test did not reveal evidence of publication bias. However, because fewer than 10 studies were included in this analysis, the assessment of publication bias may be limited (see Supplementary Materials).

### 3.7. Effect of OEP on FES-I Scores in Older Adults

**Figure 16:**
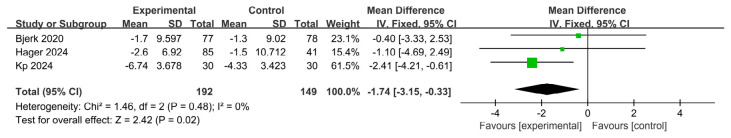
Forest plot of changes in FES-I scores [41,44,46].

A fixed-effects model was used for the analysis (Figure 16). The results suggested that OEP reduced FES-I scores among older adults (WMD = −1.74; 95%CI: −3.15, −0.33; *p* = 0.02). The certainty of evidence was rated as low, mainly due to risk of bias and limited sample size (see Supplementary Materials). Because fewer than 10 studies were included in this analysis, the assessment of publication bias may have been limited, although Egger’s test did not indicate evidence of publication bias (see Supplementary Materials).

### 3.8. Effect of OEP on 6MWT in Older Adults

**Figure 17:**
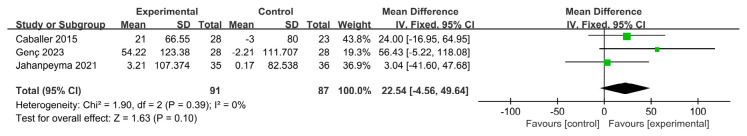
Forest plot of changes in 6MWT (meters) [40,42,43].

Analysis using a fixed-effects model showed that no statistically significant improvement in 6MWT was observed following OEP intervention among older adults (Figure 17).

## 4. Discussion

This systematic review and meta-analysis evaluated the effects of OEP on improving physical function related to the risk of falls in older adults. The results showed that OEP may improve TUG, BBS, SPPB, 30s-SST, and FES-I, but did not result in a statistically significant improvement in the 6MWT. Although several outcomes in this study reached statistical significance, the clinical relevance of these changes should be interpreted cautiously with reference to established minimal clinically important differences (MCID) or minimal detectable changes (MDC). For TUG, the combined effect in this study was −1.47 s. However, this improvement did not clearly reach the MCID or MDC thresholds reported in previous studies [62,63]. This suggests that the statistically significant improvement in TUG may not necessarily represent a clinically meaningful or truly detectable change. The results of this study suggest that OEP may significantly improve BBS scores in older adults (WMD = 4.43; 95%CI: 2.50, 6.36; *p* < 0.001). Previous studies have reported a wide range of BBS MCID values, approximately 1.9 to 24.5 points [62]. In addition, BBS MDC values also vary according to baseline balance function, ranging from approximately 4 to 7 points [64]. Although the WMD observed in this study exceeded the lower MCID and MDC thresholds, it did not clearly reach all of the higher thresholds reported in the literature. Its clinical relevance should still be interpreted cautiously because these thresholds may vary across populations and baseline functional levels. For SPPB, the WMD in this study was 0.76 points, which exceeded the small meaningful change threshold of 0.5 points reported by Perera et al. [65], but did not reach the substantial meaningful change threshold of 1.0 point. Therefore, the improvement in SPPB may have some clinical relevance, but should still be interpreted with caution. For 30s-SST and FES-I, both outcomes reached statistical significance. The WMD for 30s-SST was 2.68 repetitions, which exceeded the previously reported MCID threshold of 2 repetitions [66], suggesting possible clinical relevance. In contrast, the WMD for FES-I was −1.74 points, which was below previously reported MCID thresholds [67], suggesting limited clinical relevance. However, because these MCID values were mainly derived from specific patient populations, these findings should be interpreted with caution.

The study also used subgroup analysis to further explore the potential influence of the intervention model on OEP effects. The results showed that, although OEP was originally designed as a personalized home exercise program, our findings suggest that group-based OEP may offer superior efficacy in improving physical function related to fall risk among older adults. This finding is consistent with the results of the meta-analysis by Chiu et al. and Peng et al. [68,69]. We believe this may be related to the fact that when older adults receive in-person training and guidance, their movements are more precise, and their compliance and motivation are higher [70]. Group-based exercise may provide a better setting in which trained professionals can monitor exercise performance, provide timely guidance, and support regular attendance and adherence [30]. At the same time, the peer encouragement and sense of social interaction provided by group exercise can offer older adults greater emotional support and external motivation during training, thereby strengthening their motivation to participate, increasing the likelihood of continued training, and reducing the risk of boredom, incorrect movement execution and dropout that may occur during independent training at home [71,72,73]. Another meta-analysis also demonstrated that supervised group exercises help improve functional performance and reduce the risk of falls among older adults in the community [74].

In terms of intervention duration, interventions lasting four months or longer generally resulted in more sustained and significant improvements, whereas interventions lasting less than four months showed greater heterogeneity in outcomes. He et al. [75] also concluded that OEP lasting at least 12 weeks or longer is necessary to achieve fall prevention.

In the age-based subgroup analysis, we further compared the effectiveness of OEP among older adults of different ages. The results suggested that greater improvements in TUG and 30s-SST were observed among adults aged ≥75 years. This finding is consistent with the view, as presented in guidelines and implementation experience, that OEP offers more significant fall-prevention benefits among very elderly individuals [24,76]. From a pathophysiological perspective, the age of 75 and over is generally regarded as a clinically significant stage of advanced age. At this stage, older adults are more likely to experience degenerative changes in the neuromuscular and sensorimotor systems, such as type II fast-twitch muscle fiber atrophy, reduced muscle quality, and sensory impairment [77,78,79]. These age-related functional declines may result in lower baseline physical function, thereby providing greater potential for improvement after systematic and individualized exercise training [24]. Therefore, this population may be more likely to benefit from OEP, which focuses on lower-limb strengthening and balance training. However, as the number of studies included in some age subgroups was limited, and substantial heterogeneity remained in several analyses, the findings from the age-based subgroups should be regarded as exploratory rather than confirmatory. Significant heterogeneity was observed in several outcomes, particularly TUG, BBS, and 30s-SST. This study further explores the sources of heterogeneity through subgroup analysis and leave-one-out sensitivity analyses. In the TUG outcome measures, heterogeneity decreased significantly after excluding the study by Zou et al. [50], whilst the direction and statistical significance of the overall meta-analysis results remained unchanged, suggesting that this study may be the primary source of heterogeneity in the TUG analysis. The participants in the study by Zou et al. [50] had the highest mean age among the included studies. Compared with populations in other studies, they may have had lower baseline physical function, more pronounced impairments in lower-limb strength and balance control, and greater potential for improvement. Therefore, OEP may have produced more pronounced improvements in TUG performance in this study population. In addition, the risk-of-bias assessment indicated uncertainty regarding random sequence generation and allocation concealment, as well as a high risk of performance bias. These clinical and methodological differences may partly explain why the study by Zou et al. [50] was a major contributor to heterogeneity in the TUG analysis.

In contrast, for the BBS and 30s-SST, excluding each study in turn did not change the direction or statistical significance of the overall meta-analytic results. However, heterogeneity remained high after each exclusion. This suggests that the heterogeneity was not driven by a single study. Instead, it may be related to differences between studies, including baseline participant characteristics, control-group interventions, intervention duration, frequency, and intervention model [80]. In some studies, the control interventions were not clearly or consistently described, which may have contributed to heterogeneity. Meta-regression was not performed because the number of studies included for each outcome was limited, and the results may have been unstable.

It is worth noting that the study did not find that OEP had a significant positive effect on the 6MWT. In the study by Wang et al. [81], although a significant overall improvement in the 6MWT was reported, their subgroup analysis indicated that the beneficial effects were primarily observed in older adults with impaired health, whereas healthy older adults did not show significant improvement. We believe this is related to the fact that the OEP primarily focuses on strength and balance rather than cardiorespiratory endurance. OEP can improve lower-body strength and balance, thereby supporting daily functional tasks required for basic activities of daily living, such as transitioning between sitting and standing and moving around indoors. The 6MWT reflects long-distance walking ability and overall endurance, whereas OEPs include relatively few training components targeting cardiorespiratory endurance or sustained walking ability, resulting in a relatively limited effect on 6MWT performance. In fact, low-to-moderate-intensity functional training has limited effects on improving cardiorespiratory endurance, whereas moderate-to-high-intensity aerobic training demonstrates greater benefits in the 6MWT [82,83].

To our knowledge, few meta-analyses comprehensively evaluate the effects of OEP on physical function outcomes related to fall risk in older adults, and evidence on the optimization of OEP implementation strategies remains limited. This study conducted a meta-analysis on the effects of OEP on physical function related to fall risk in older adults and compared the outcomes across different age groups and implementation models. Unlike previous studies that focused solely on the overall effectiveness of OEP, this study found that even with identical training content, optimizing the implementation strategy can significantly influence intervention outcomes. We also note that the small sample sizes for some outcomes in this study may affect the reliability of the effect sizes, and further research is needed.

A limitation of our meta-analysis is the substantial heterogeneity observed in several outcomes (e.g., TUG, BBS, 30s-SST). Although subgroup analyses and sensitivity analyses by excluding each study in turn were performed, some heterogeneity remained unexplained. This may be related to substantial differences in participant characteristics across studies, as well as variations in control conditions and other factors. Some further limitations of the present study also need to be acknowledged. Some outcomes were based on a limited number of studies (e.g., SPPB, FES-I, 6MWT). This may reduce the statistical power and accuracy of the relevant results. Although Egger’s test did not show clear evidence of publication bias, potential publication bias should still be considered because only a small number of studies were included for several outcomes. In addition, this study only included peer-reviewed studies published in English, which may have introduced language bias. According to the GRADE assessment, the certainty of evidence for all outcomes was rated as low.

As more standardized studies are conducted in the future, the issues of small sample sizes and high heterogeneity in results will be addressed. We also suggest that future research explores whether video-assisted technologies can overcome the limitations of home-based intervention models.

## 5. Conclusions

Evidence from this systematic review and meta-analysis suggests that OEP may improve physical function performance related to fall risk in older adults, such as the TUG, BBS, SPPB, 30s-SST, and FES-I, but has no effect on the 6MWT. A group-based OEP lasting at least 4 months appeared to show more favorable effects among older adults (especially adults aged ≥75 years).

## Figures and Tables

**Table 1:** Characteristics of the studies included.

Study and Year	Participants		Age		Intervention		Duration (mo)	Frequency (t/wk)	Mode	Outcomes
	OEP	Control	OEP	Control	OEP	Control				
Caballer et al. (2016) [40]	28	23	69.1 ± 4	69.0 ± 3.3	OEP	Usual care	4	3	Group-based	①↓②↑③↑⑥↑
Bjerk et al. (2020) [41]	77	78	83.1 ± 6.7	82.2 ± 6.7	OEP	Usual care	3	3	Home-based	②↑④↑⑤↓
Genç et al. (2023) [42]	28	28	74.38 ± 5.24	73.75 ± 6.8	OEP	Usual care	3	3	Group-based	③↑④↑⑥↑
Jahanpeyma et al. (2021) [43]	35	36	74.6 ± 5.9	75.8 ± 4.5	OEP	Usual care	3	3	Group-based	②↑④↑⑥↑
Hager et al. (2024) [44]	85	41	79 ± 6.6	80 ± 7.6	OEP	Usual care	3	3	Home-based	①↓③↑⑤↓
Iliffe et al. (2014) [45]	178	210	≥65	≥65	OEP	Usual care	3	3	Home-based	①↓④↑
Kp et al. (2024) [46]	30	30	67.6 ± 1.81	67.47 ± 1.73	OEP	Gaze stability exercises	2	3	Home-based	②↑⑤↓
Liew et al. (2019) [47]	24	24	75.9 ± 7.0	75.2 ± 7.2	OEP	Usual care	6	3	Home-based	①↓
Ambrose et al. (2008) [27]	28	24	81.4 ± 6.2	83.1 ± 6.3	OEP	Usual care	6	3	Group-based	①↓
Santos et al. (2023) [48]	12	12	83 ± 4.65	84.92 ± 3.85	OEP	Usual care	3	2	Group-based	①↓
Kang et al. (2024) [49]	31	9	79.8 ± 3.5	81.4 ± 3.7	OEP	Usual care	6	2	Group-based	①↓③↑④↑
Zou et al. (2022) [50]	29	28	85.17 ± 3.1	85.79 ± 5.06	OEP	Usual care	3	2	Group-based	①↓④↑
Lytras et al. (2022) [51]	75	75	70 ± 1.5	70 ± 1.5	OEP	Usual care	6	2	Group-based	①↓②↑④↑

① Timed Up and Go test, TUG; ② Berg Balance Scale, BBS; ③ Short Physical Performance Battery, SPPB; ④ 30-Second Sit-to-Stand Test, 30s-SST; ⑤ Falls Efficacy Scale-International, FES-I; ⑥ Six-Minute Walk Test, 6MWT; ↑: In this study, this indicator showed an upward trend. ↓: In this study, this indicator showed a downward trend.

## Data Availability

Extracted data are available upon reasonable request to the corresponding authors.

## Supplementary Materials

The following supporting information can be downloaded at https://www.mdpi.com/article/10.3390/healthcare14142187/s1. Figure S1: Sensitivity analysis; Figure S2: Funnel plot; Figure S3: Egger’s test; Table S1: PRISMA 2020 Checklist of items; Table S2: Search Terms; Table S3: Egger’s test; Table S4: Leave-one-out sensitivity analysis table; Table S5: The certainty of the evidence.
