# Supplementary material for: Effects of the Otago Exercise Program on Physical Function Performance Related to Falls in the Elderly: A Meta-Analysis of Randomized Controlled Trials
Source: Healthcare (Basel). 2026 Jul 20;14(14):2187. doi: 10.3390/healthcare14142187 (PMC13411888; doi:10.3390/healthcare14142187)
Supplement: Supplementary file 1 [file healthcare-14-02187-s001.zip › healthcare-4326603-supplementary.pdf]

Supplementary Material

Effects of the Otago Exercise Program on Physical Function Performance Related to the Risk of Falls in the Elderly: A Meta-analysis of Randomized Controlled Trials

Contents

Table S1. PRISMA 2020 Checklist of items 2

Table S2.Search Terms 6

Figure S1.Sensitivity analysis 7

Figure S2.Funnel plot 10

Figure S3.Egger’s test 13

Table S3. Egger's test 16

Table S4. Leave-one-out sensitivity analysis table 17

Table S5. The certainty of the evidence 19

Table S1. PRISMA 2020 Checklist of items

Section and Topic	Item #	Checklist item	Location where item is reported
**TITLE**			
Title	1	Identify the report as a systematic review.	Page 1
**ABSTRACT**			
Abstract	2	See the PRISMA 2020 for Abstracts checklist.	Page 1-2
**INTRODUCTION**			
Rationale	3	Describe the rationale for the review in the context of existing knowledge.	Page 2-3
Objectives	4	Provide an explicit statement of the objective(s) or question(s) the review addresses.	Page 2-3
**METHODS**			
Eligibility criteria	5	Specify the inclusion and exclusion criteria for the review and how studies were grouped for the syntheses.	Page 3-4
Information sources	6	Specify all databases, registers, websites, organisations, reference lists and other sources searched or consulted to identify studies. Specify the date when each source was last searched or consulted.	Page 3
Search strategy	7	Present the full search strategies for all databases, registers and websites, including any filters and limits used.	Page 3<br>Supplementary Material
Selection process	8	Specify the methods used to decide whether a study met the inclusion criteria of the review, including how many reviewers screened each record and each report retrieved, whether they worked independently, and if applicable, details of automation tools used in the process.	Page 3
Data collection process	9	Specify the methods used to collect data from reports, including how many reviewers collected data from each report, whether they worked independently, any processes for obtaining or confirming data from study investigators, and if applicable, details of automation tools used in the	Page 4-5

Section and Topic	Item #	Checklist item	Location where item is reported
		process.	
Data items	10a	List and define all outcomes for which data were sought. Specify whether all results that were compatible with each outcome domain in each study were sought (e.g. for all measures, time points, analyses), and if not, the methods used to decide which results to collect.	Page 4-5
	10b	List and define all other variables for which data were sought (e.g. participant and intervention characteristics, funding sources). Describe any assumptions made about any missing or unclear information.	Page 3-5
Study risk of bias assessment	11	Specify the methods used to assess risk of bias in the included studies, including details of the tool(s) used, how many reviewers assessed each study and whether they worked independently, and if applicable, details of automation tools used in the process.	Page 4
Effect measures	12	Specify for each outcome the effect measure(s) (e.g. risk ratio, mean difference) used in the synthesis or presentation of results.	Page 5
Synthesis methods	13a	Describe the processes used to decide which studies were eligible for each synthesis (e.g. tabulating the study intervention characteristics and comparing against the planned groups for each synthesis (item #5)).	Page 4-5
	13b	Describe any methods required to prepare the data for presentation or synthesis, such as handling of missing summary statistics, or data conversions.	Page 5
	13c	Describe any methods used to tabulate or visually display results of individual studies and syntheses.	Page 5
	13d	Describe any methods used to synthesize results and provide a rationale for the choice(s). If meta-analysis was performed, describe the model(s), method(s) to identify the presence and extent of statistical heterogeneity, and software package(s) used.	Page 5
	13e	Describe any methods used to explore possible causes of heterogeneity among study results (e.g. subgroup analysis, meta-regression).	Page 4-5
	13f	Describe any sensitivity analyses conducted to assess robustness of the synthesized results.	Page 5
Reporting bias assessment	14	Describe any methods used to assess risk of bias due to missing results in a synthesis (arising from reporting biases).	Page 5
Certainty assessment	15	Describe any methods used to assess certainty (or confidence) in the body of evidence for an outcome.	Page 4

Section and Topic	Item #	Checklist item	Location where item is reported
**RESULTS**			
Study selection	16a	Describe the results of the search and selection process, from the number of records identified in the search to the number of studies included in the review, ideally using a flow diagram.	Page 4-5
	16b	Cite studies that might appear to meet the inclusion criteria, but which were excluded, and explain why they were excluded.	Page 4-5
Study characteristics	17	Cite each included study and present its characteristics.	Page 6
Risk of bias in studies	18	Present assessments of risk of bias for each included study.	Page 8-7
Results of individual studies	19	For all outcomes, present, for each study: (a) summary statistics for each group (where appropriate) and (b) an effect estimate and its precision (e.g. confidence/credible interval), ideally using structured tables or plots.	Page 7-12
Results of syntheses	20a	For each synthesis, briefly summarise the characteristics and risk of bias among contributing studies.	Page 7-12
	20b	Present results of all statistical syntheses conducted. If meta-analysis was done, present for each the summary estimate and its precision (e.g. confidence/credible interval) and measures of statistical heterogeneity. If comparing groups, describe the direction of the effect.	Page 7-12
	20c	Present results of all investigations of possible causes of heterogeneity among study results.	Page 7-12
	20d	Present results of all sensitivity analyses conducted to assess the robustness of the synthesized results.	Page 7-12
Reporting biases	21	Present assessments of risk of bias due to missing results (arising from reporting biases) for each synthesis assessed.	Page 7-12
Certainty of evidence	22	Present assessments of certainty (or confidence) in the body of evidence for each outcome assessed.	Page 7-12
**DISCUSSION**			
Discussion	23a	Provide a general interpretation of the results in the context of other evidence.	Page 12-13

Section and Topic	Item #	Checklist item	Location where item is reported
	23b	Discuss any limitations of the evidence included in the review.	Page 12-13
	23c	Discuss any limitations of the review processes used.	Page 12-13
	23d	Discuss implications of the results for practice, policy, and future research.	Page 12-13
**OTHER INFORMATION**			
Registration and protocol	24a	Provide registration information for the review, including register name and registration number, or state that the review was not registered.	Page 3
	24b	Indicate where the review protocol can be accessed, or state that a protocol was not prepared.	Page 3
	24c	Describe and explain any amendments to information provided at registration or in the protocol.	Page 3
Support	25	Describe sources of financial or non-financial support for the review, and the role of the funders or sponsors in the review.	Page 14
Competing interests	26	Declare any competing interests of review authors.	Page 14
Availability of data, code and other materials	27	Report which of the following are publicly available and where they can be found: template data collection forms; data extracted from included studies; data used for all analyses; analytic code; any other materials used in the review.	Supplementary Material

Table S2.Search Terms

Supplementary table 2. Search equations according to PICOS question

Databases	Search equations
Pubmed	(((((Otago Exercise Programme[Title/Abstract]) OR (Otago exercise[Title/Abstract])) OR (OEP[Title/Abstract])) OR (otago[Title/Abstract])) AND (("Aged"[Mesh]) OR (Elderly[Title/Abstract]))) AND (((((((("Accidental Falls"[Mesh]) OR (Fall[Title/Abstract])) OR (Falls, Accidental[Title/Abstract])) OR (Accidental Fall[Title/Abstract])) OR (Fall, Accidental[Title/Abstract])) OR (Falling[Title/Abstract])) OR (Falls[Title/Abstract])) OR (Slip[Title/Abstract] AND Fall[Title/Abstract])) OR (Fall[Title/Abstract] AND Slip[Title/Abstract]))
WOS	(TS=(otago) OR AB=(otago OR Otago Exercise Programme OR Otago exercise OR OEP)) AND (TS=(Accidental Falls) OR AB=(Accidental Falls OR Falls, Accidental OR Accidental Fall OR Fall, Accidental OR Falling OR Falls OR Slip and Fall OR Fall and Slip)) AND (TS=(Aged) OR AB=(Aged OR Elderly))
Embase	otago:ab,ti OR 'otago exercise programme':ab,ti OR 'otago exercise':ab,ti OR oep:ab,ti<br>('accidental falls':ab,ti OR 'falls, accidental':ab,ti OR 'accidental fall':ab,ti OR 'fall, accidental':ab,ti OR falling:ab,ti OR fall:ab,ti OR falls:ab,ti OR slip:ab,ti) AND fall:ab,ti
Cochrane Library	#18 MeSH descriptor: [Accidental Falls] explode all trees 2349<br>#19 (Accidental Falls OR Falls, Accidental OR Accidental Fall OR Fall, Accidental OR Falling OR fall OR Falls OR Slip and Fall OR Fall and Slip):ti,ab,kw (Word variations have been searched) 28372<br>#20 #18 or #19 28372<br>#21 MeSH descriptor: [Aged] explode all trees 288786<br>#22 (Aged OR Elderly):ti,ab,kw (Word variations have been searched) 1039726<br>#23 #21 or #22 1039726<br>#24 (otago OR Otago Exercise Programme OR Otago exercise OR OEP):ti,ab,kw (Word variations have been searched) 490<br>#25 #24 and #23 and #20 214

Figure S1.Sensitivity analysis

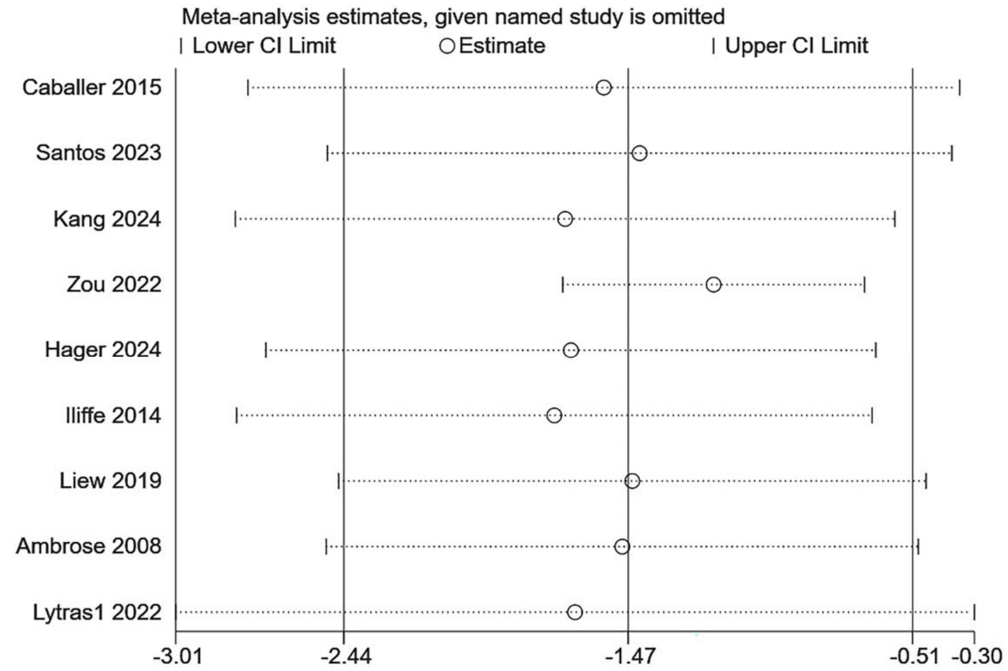

Figure S1a. Sensitivity analysis of TUG

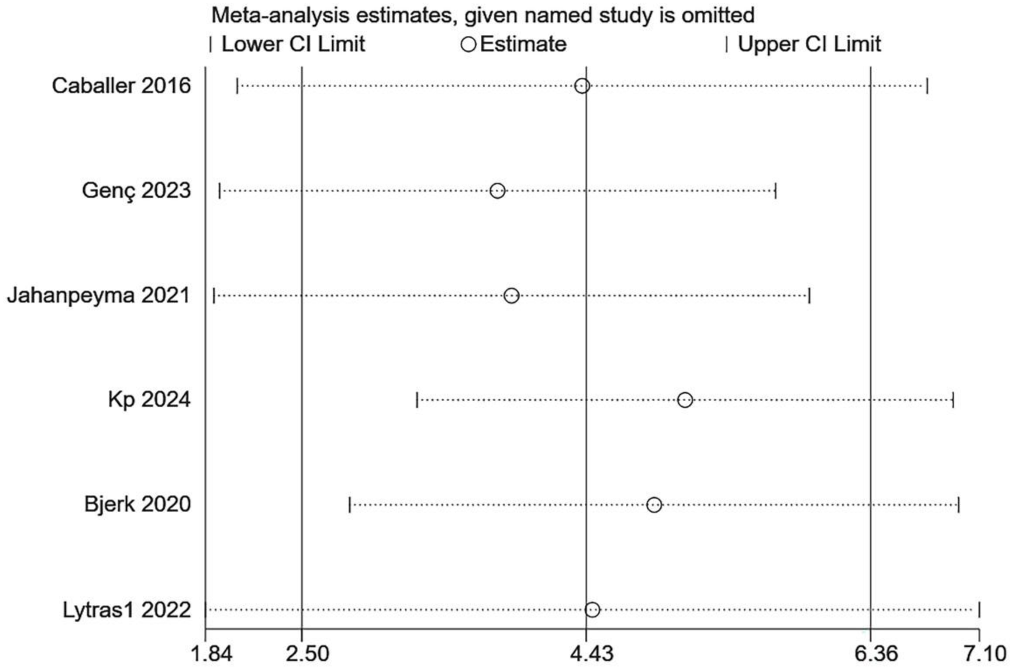

Figure S1b. Sensitivity analysis of BBS

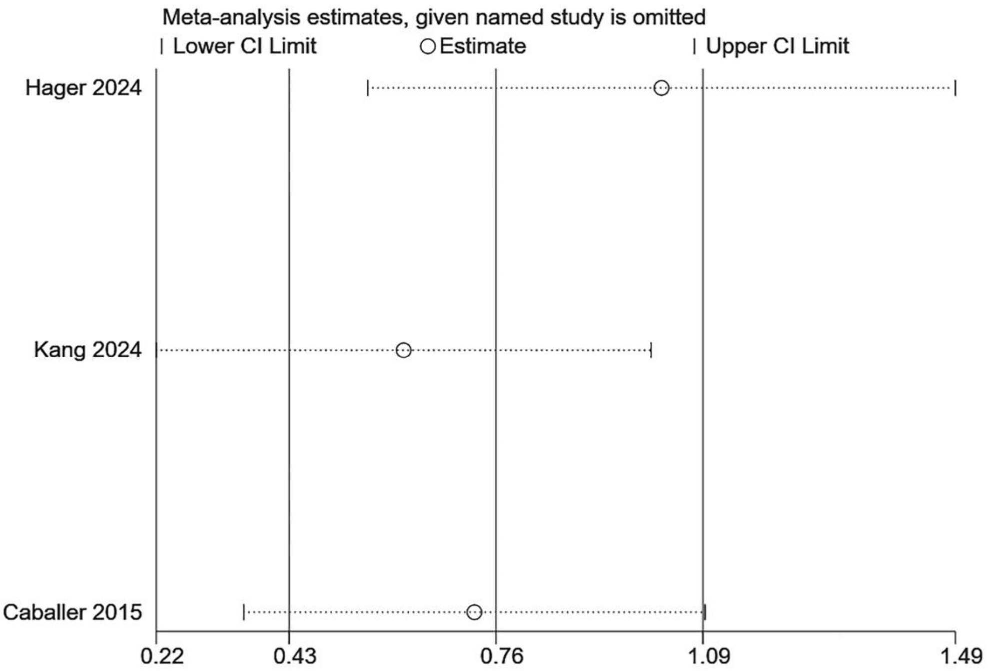

Figure S1c. Sensitivity analysis of SPPB

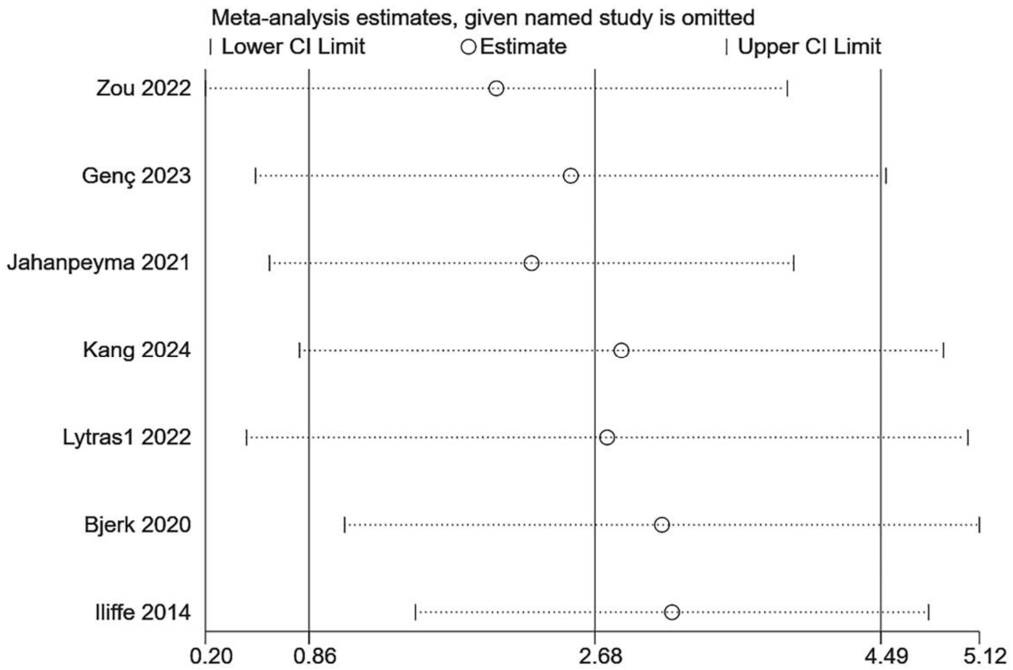

Figure S1d. Sensitivity analysis of 30s-SST

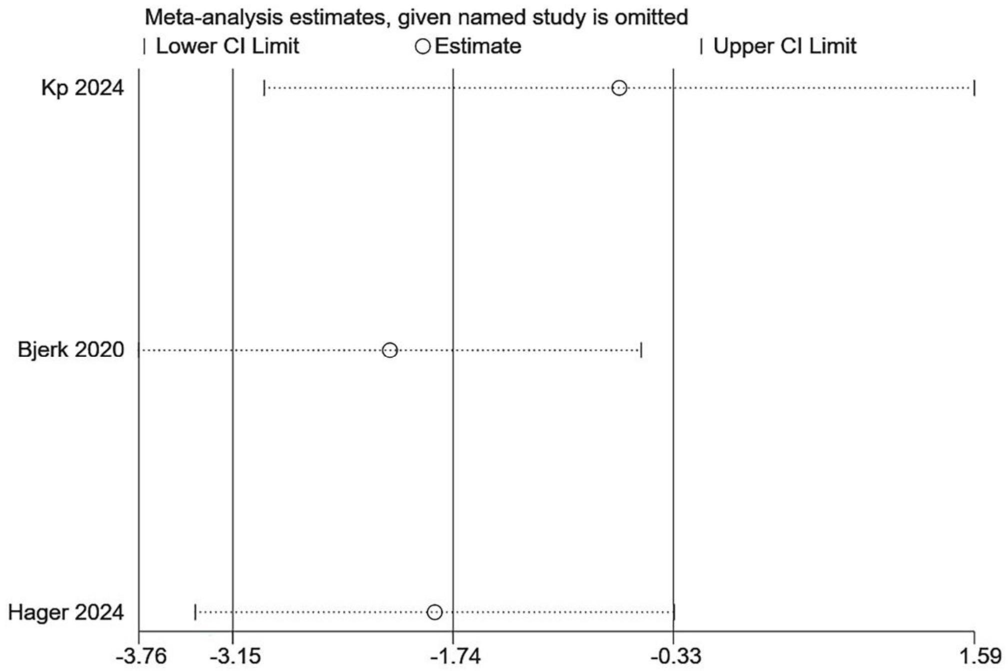

Figure S1e. Sensitivity analysis of FES-I

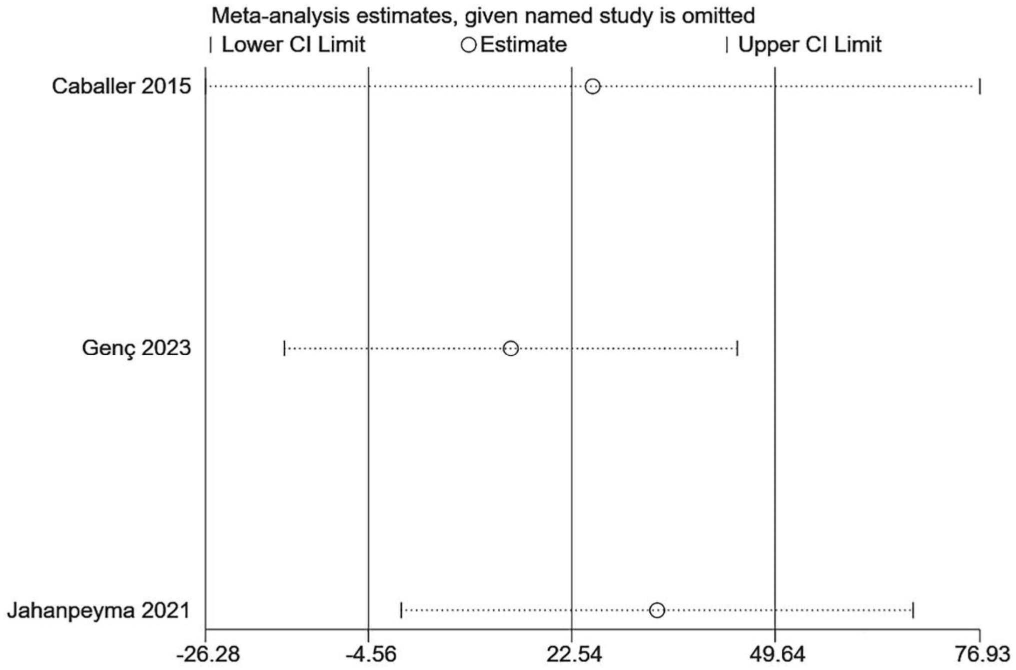

Figure S1f. Sensitivity analysis of 6MWT

Figure S2.Funnel plot

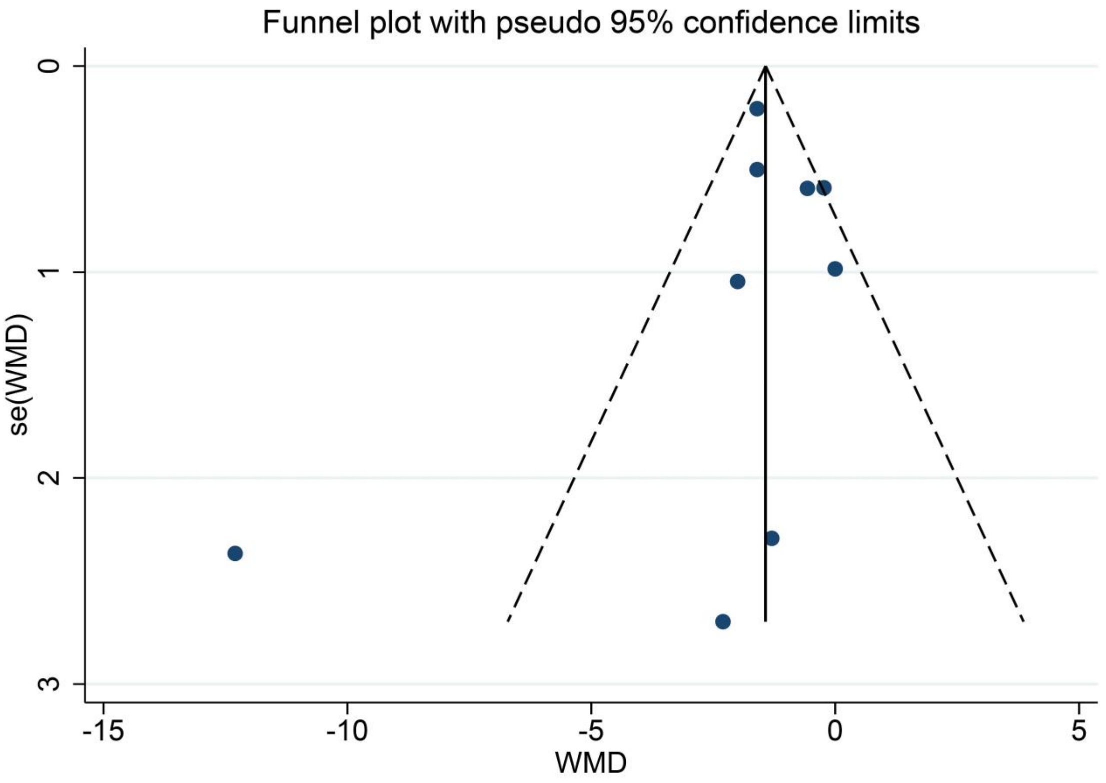

Figure S2a. Funnel plot of TUG

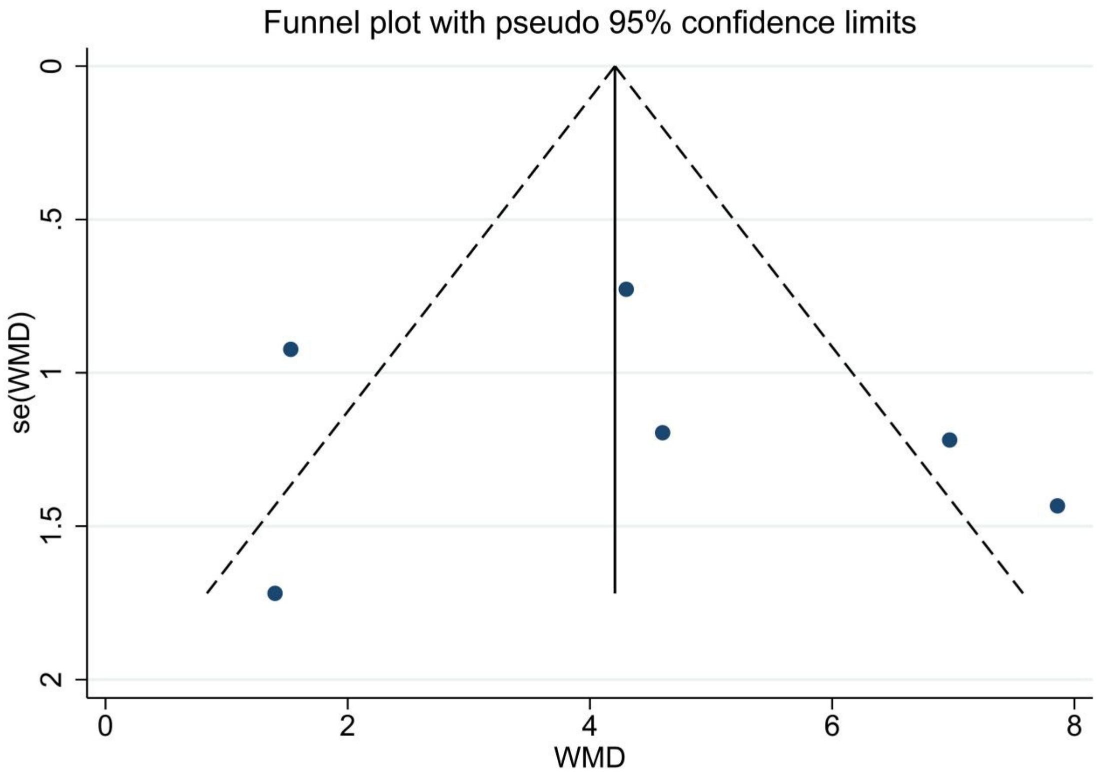

Figure S2b. Funnel plot of BBS

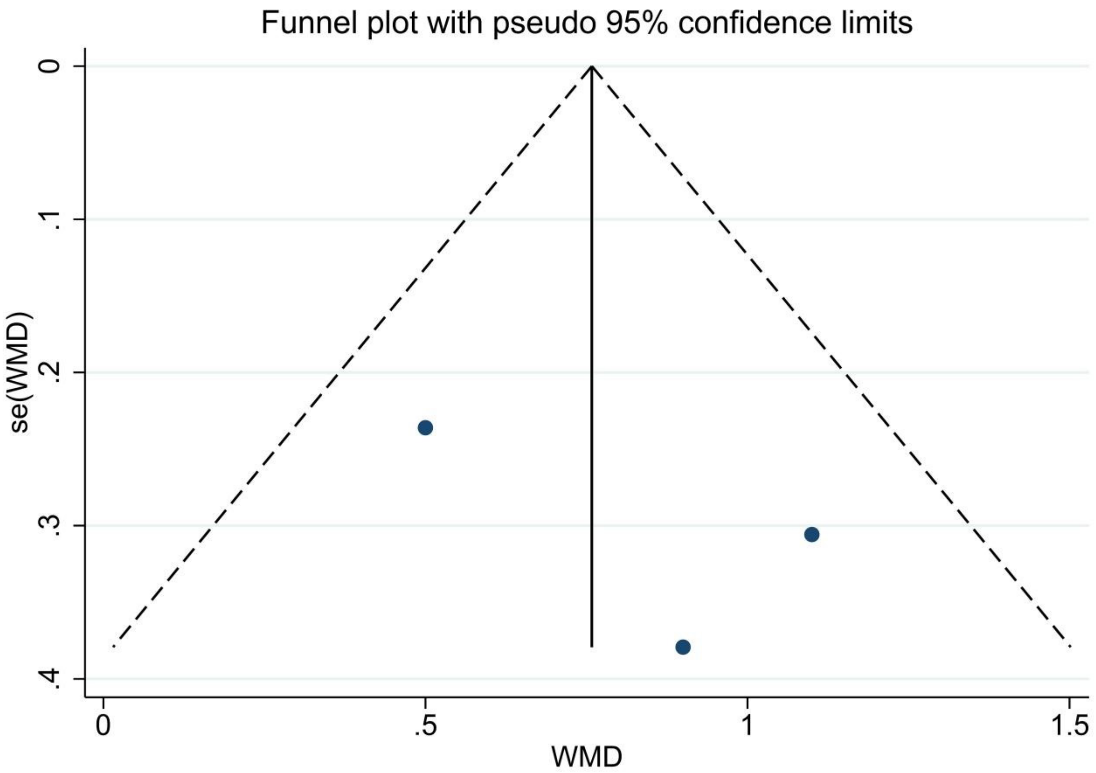

Figure S2c. Funnel plot of SPPB

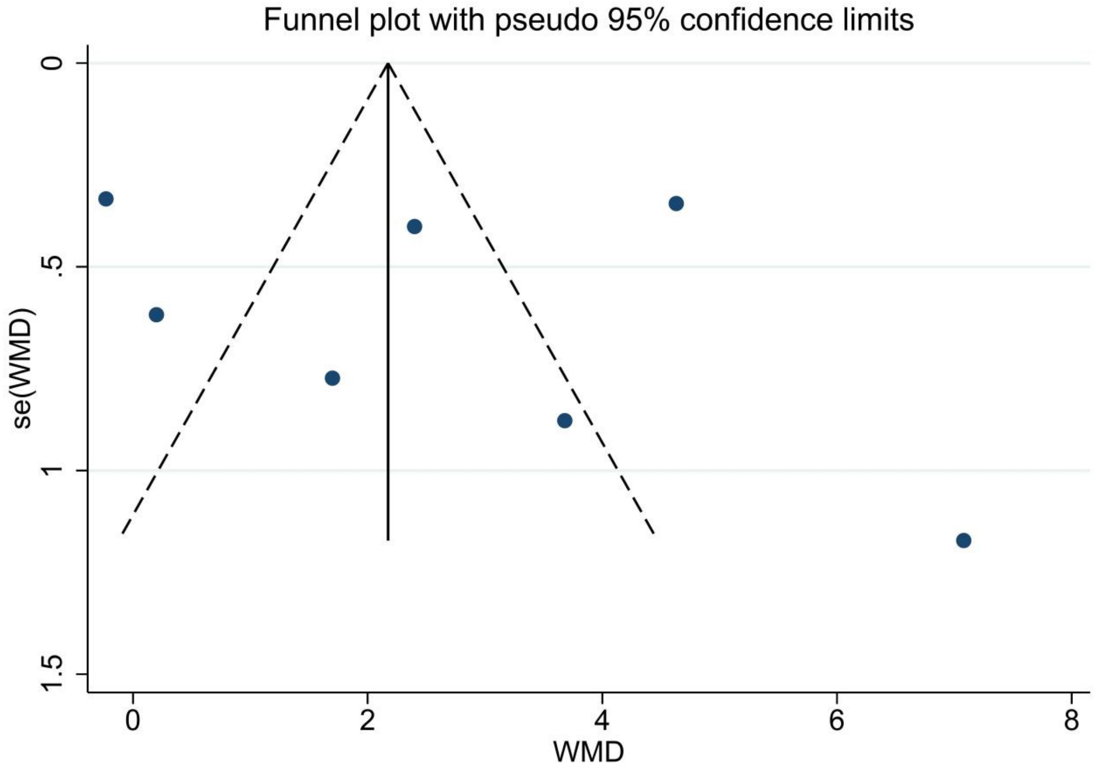

Figure S2d. Funnel plot of 30s-SST

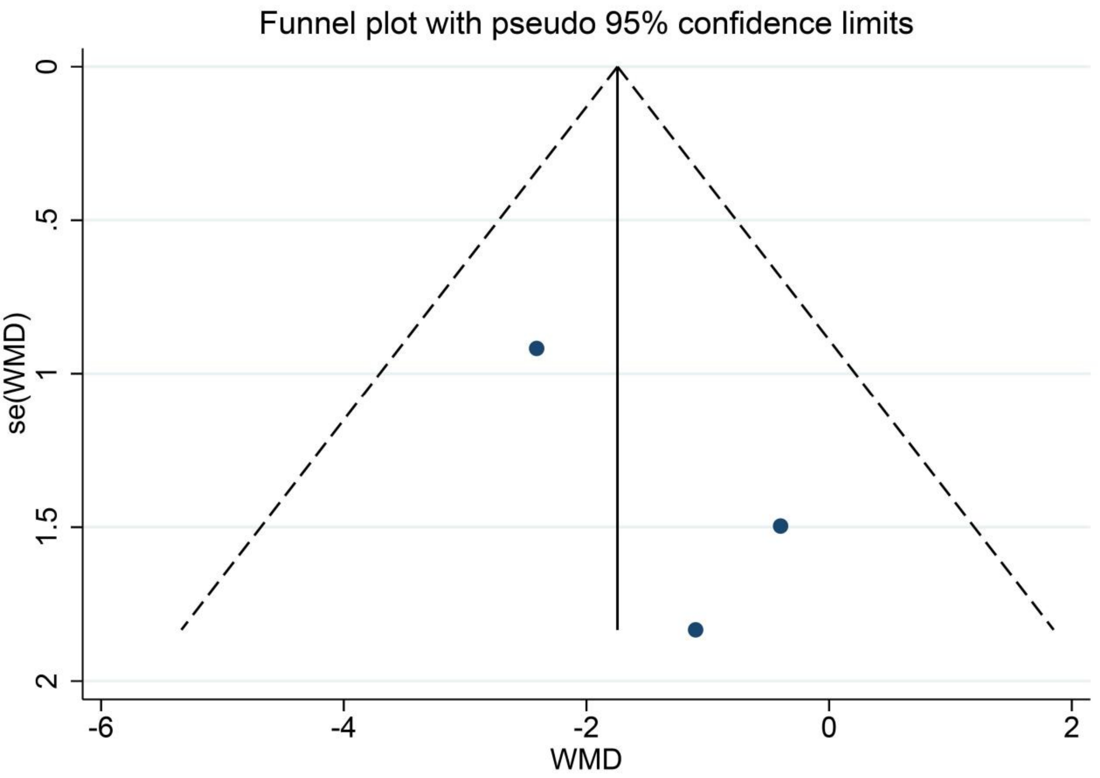

Figure S2e. Funnel plot of FES-I

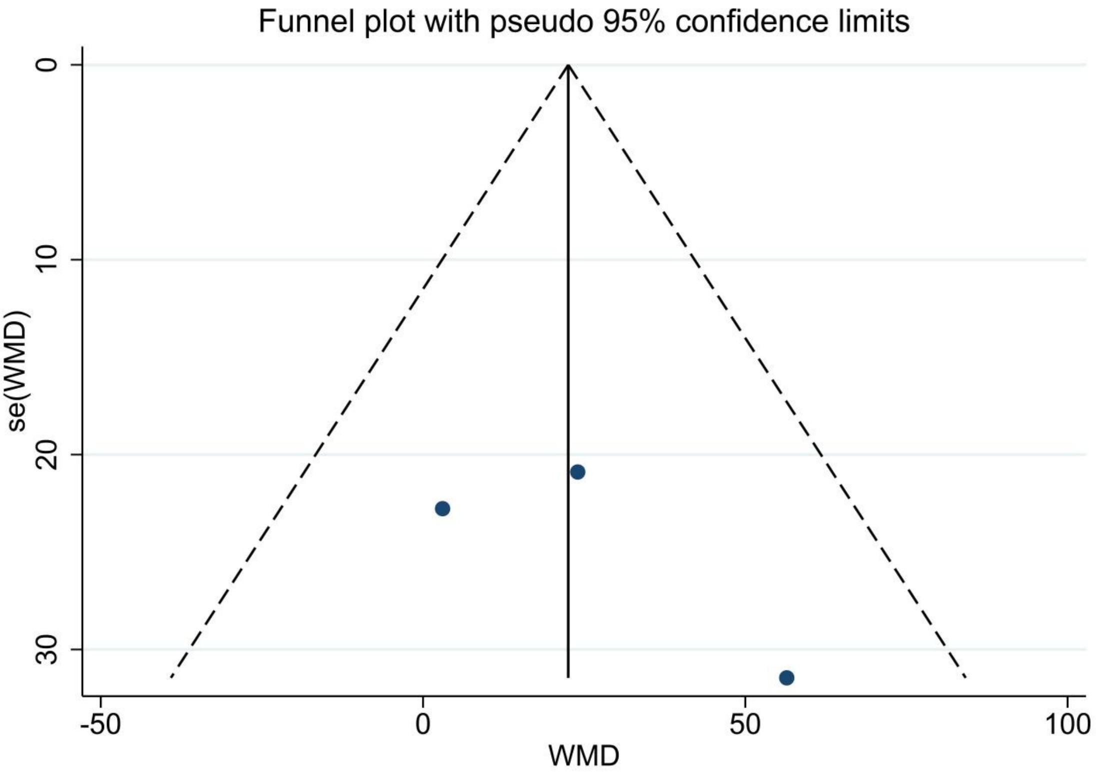

Figure S2f. Funnel plot of 6MWT

Figure S3.Egger's test

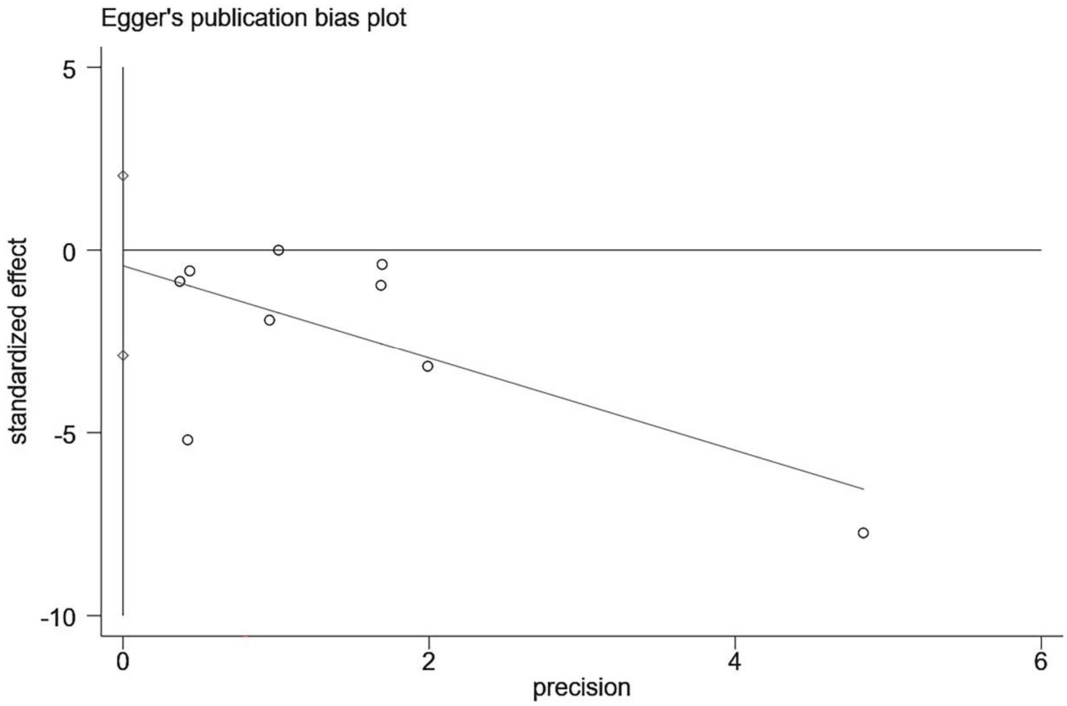

Figure S3a. Egger's test of TUG

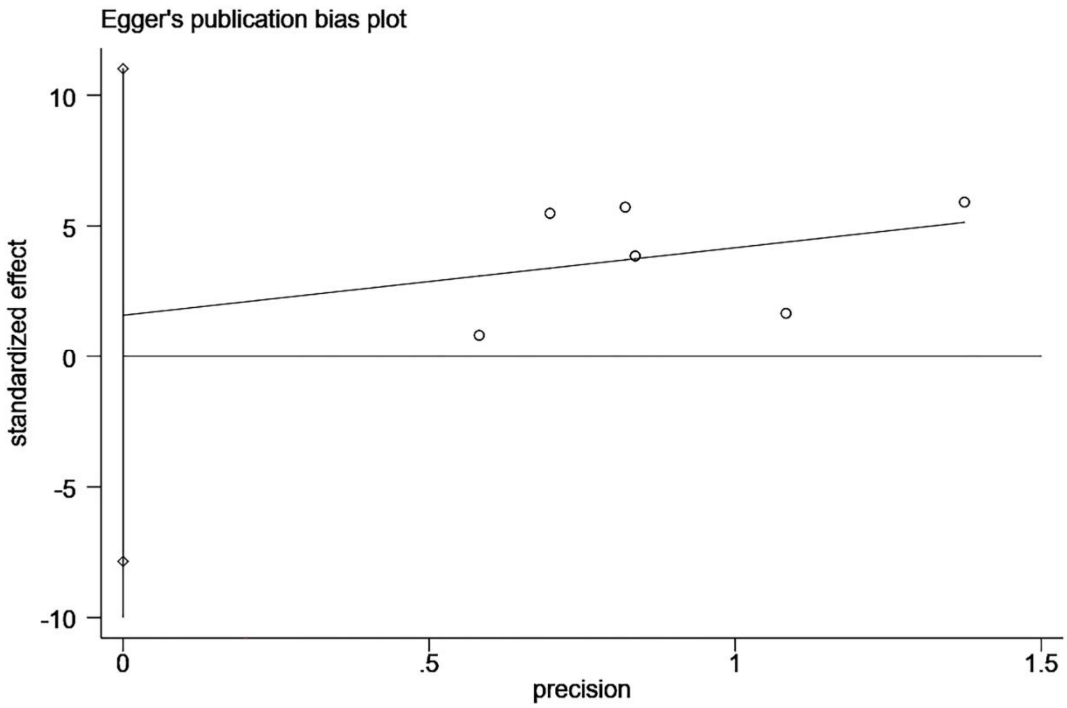

Figure S3b. Egger's test of BBS

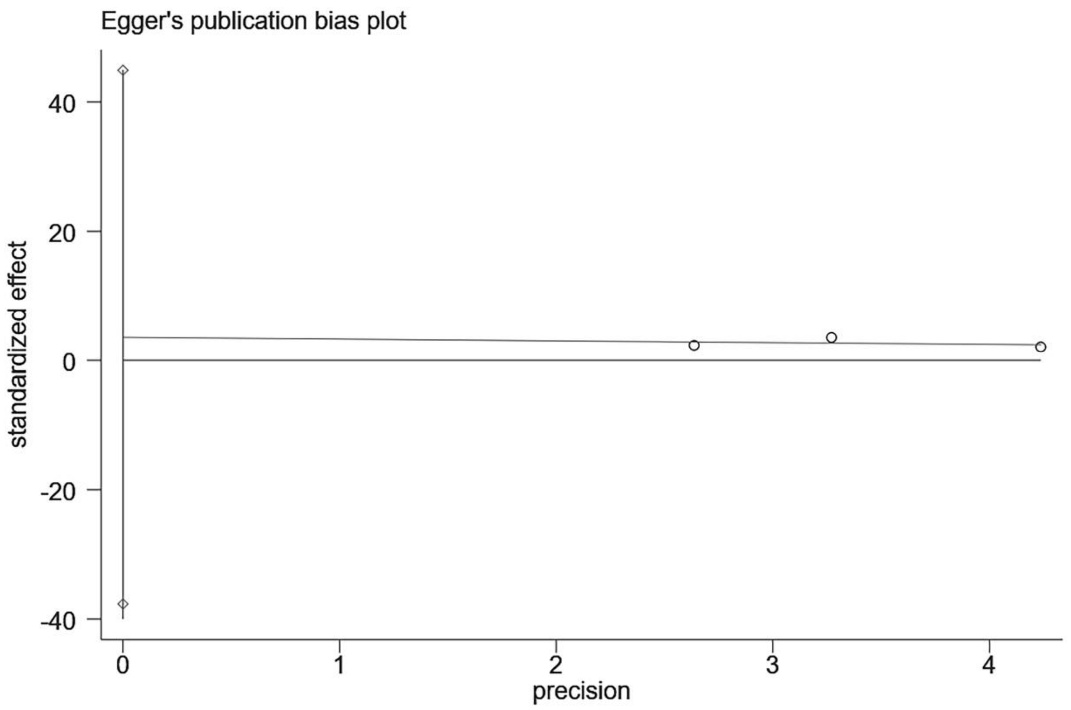

Figure S3c. Egger’s test of SPPB

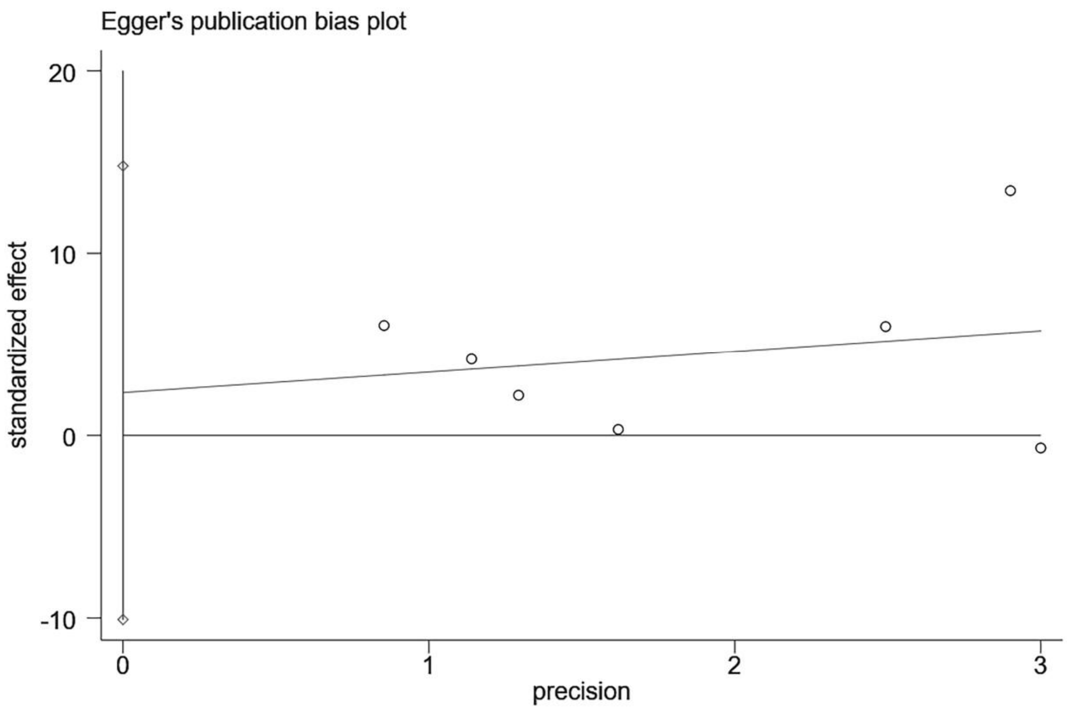

Figure S3d. Egger’s test of 30s-SST

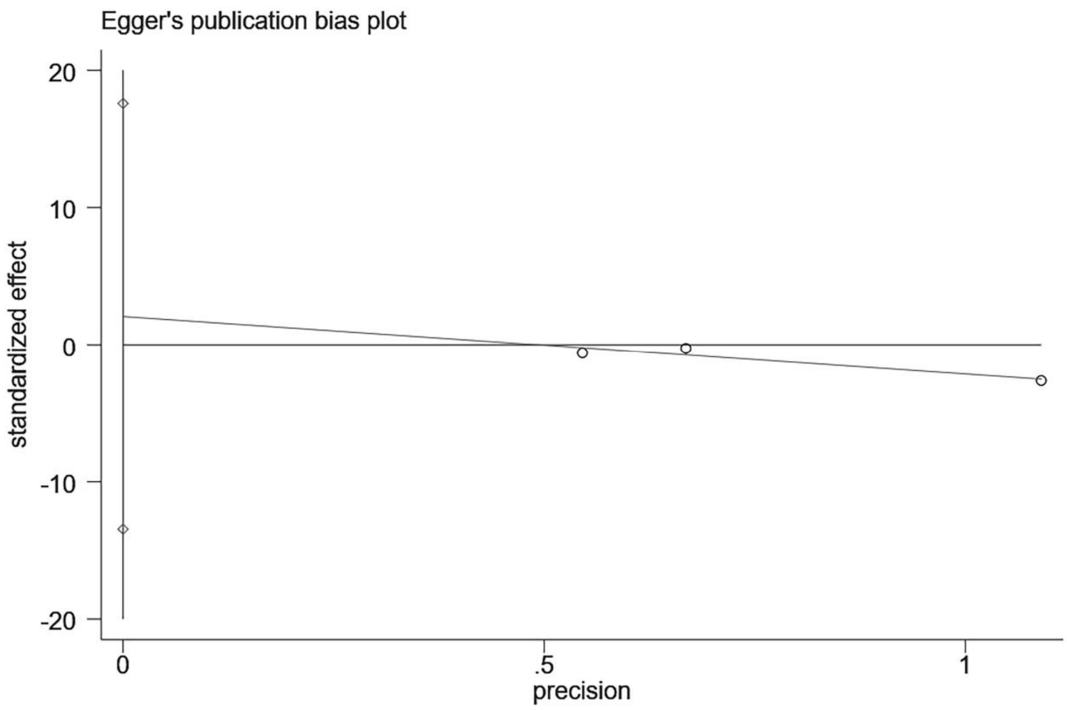

Figure S3e. Egger's test of FES-I

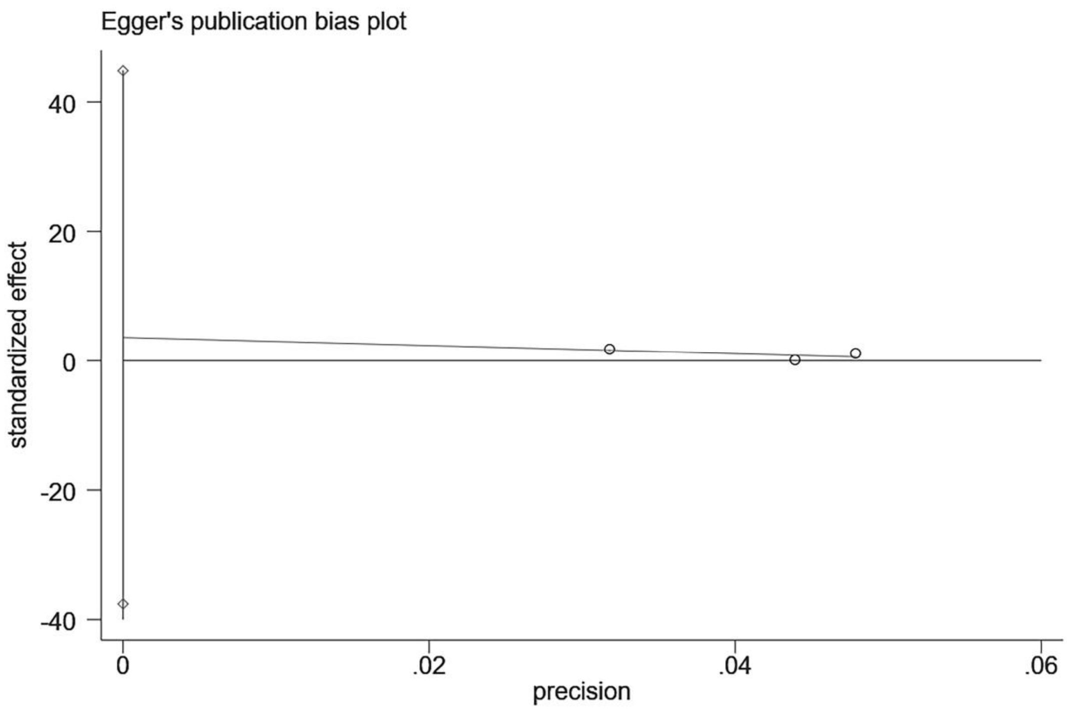

Figure S3f. Egger's test of 6MWT

Table S3. Egger's test

Table S3a. Egger’s test of TUG

Std_Eff	Coef.	Std. Err.	t	P>|t|	[95% Conf. Interval]	
slope	-1.264105	.5242127	-2.41	0.047	-2.503671	-.0245386
bias	-.428331	1.041471	-0.41	0.693	-2.891019	2.034357

Table S3b. Egger’s test of BBS

Std_Eff	Coef.	Std. Err.	t	P>|t|	[95% Conf. Interval]	
slope	2.585801	3.628247	0.71	0.515	-7.487828	12.65943
bias	1.580647	3.397298	0.47	0.666	-7.851764	11.01306

Table S3c. Egger’s test of SPPB

Std_Eff	Coef.	Std. Err.	t	P>|t|	[95% Conf. Interval]	
slope	-.2722638	.9432007	-0.29	0.821	-12.25677	11.71224
bias	3.617095	3.249174	1.11	0.466	-37.66758	44.90177

Table S3d. Egger’s test of 30s-SST

Std_Eff	Coef.	Std. Err.	t	P>|t|	[95% Conf. Interval]	
slope	1.131403	2.338585	0.48	0.649	-4.880122	7.142928
bias	2.347944	4.837637	0.49	0.648	-10.0876	14.78349

Table S3e. Egger’s test of FES-I

Std_Eff	Coef.	Std. Err.	t	P>|t|	[95% Conf. Interval]	
slope	-4.203663	1.521692	-2.76	0.221	-23.5386	15.13127
bias	2.063285	1.221276	1.69	0.340	-13.4545	17.58107

Table S3f. Egger’s test of 6MWT

Std_Eff	Coef.	Std. Err.	t	P>|t|	[95% Conf. Interval]	
slope	-62.90757	77.67386	-0.81	0.567	-1049.847	924.0324
bias	3.616636	3.243195	1.12	0.465	-37.59206	44.82533

Table S4. Leave-one-out sensitivity analysis table

Table S4a. Leave-one-out sensitivity analysis for TUG

Outcome	Excluded study	WMD	95% CI	P value	I^2
TUG	None	-1.47	-2.44, -0.51	0.003	74%
TUG	Ambrose 2008	-1.49	-2.50, -0.49	0.003	77%
TUG	Caballer 2015	-1.56	-2.76, -0.35	0.01	77%
TUG	Hager 2024	-1.67	-2.70, -0.64	0.002	75%
TUG	Iliffe 2014	-1.72	-2.80, -0.65	0.002	73%
TUG	Kang 2024	-1.69	-2.80, -0.57	0.003	75%
TUG	Liew 2019	-1.46	-2.45, -0.47	0.004	77%
TUG	Lytrasl 2022	-1.65	-3.01, -0.30	0.02	75%
TUG	Santos 2023	-1.44	-2.49, -0.38	0.008	77%
TUG	Zou 2022	-1.19	-1.70, -0.67	<0.00001	26%

Table S4b. Leave-one-out sensitivity analysis for BBS

Outcome	Excluded study	WMD	95% CI	P value	I^2
BBS	None	4.43	2.50, 6.36	<0.00001	78%
BBS	Bjerk 2020	4.89	2.83, 6.96	<0.00001	80%
BBS	Caballer 2016	4.40	2.06, 6.75	0.00002	82%
BBS	Genç 2023	3.83	1.94, 5.72	<0.0001	74%
BBS	Jahanpeyma 2021	3.92	1.90, 5.95	0.0001	76%
BBS	Kp 2024	5.10	3.28, 6.92	<0.00001	67%
BBS	Lytrasl 2022	4.47	1.84, 7.10	0.0009	82%

Table S4c. Leave-one-out sensitivity analysis for 30s-sst

Outcome	Excluded study	WMD	95% CI	P value	I^2
30s-sst	None	2.68	0.86, 4.49	0.004	96%
30s-sst	Bjerk 2020	3.10	1.09, 5.12	0.003	96%
30s-sst	Genç 2023	2.52	0.52, 4.52	0.01	96%
30s-sst	Iliffe 2014	3.17	1.54, 4.80	0.0001	92%
30s-sst	Jahanpeyma 2021	2.28	0.61, 3.94	0.007	92%
30s-sst	Kang 2024	2.85	0.80, 4.89	0.006	96%
30s-sst	Lytrasl 2022	2.75	0.47, 5.04	0.02	96%
30s-sst	Zou 2022	2.05	0.20, 3.90	0.03	96%

Table S5. The certainty of the evidence

Certainty assessment							№ of patients		Effect		Certainty	Importance
№ of studies	Study design	Risk of bias	Inconsistency	Indirectness	Imprecision	Other considerations	OEP	routine care	Relative (95% CI)	Absolute (95% CI)		
**TUG**												
9	randomised trials	serious[a]	serious[b]	not serious	not serious	none	490	446	-	MD **1.47 higher** (0.51 higher to 2.44 higher)	⨁⨁◯◯ Low[a,b]	CRITICAL
**BBS**												
6	randomised trials	serious[a]	serious[b]	not serious	not serious	none	273	270	-	MD **4.43 higher** (2.5 higher to 6.36 higher)	⨁⨁◯◯ Low[a,b]	CRITICAL
**SPPB**												
3	randomised trials	serious[a]	not serious	not serious	serious[c]	none	144	73	-	MD **0.76 higher** (0.43 higher to 1.09 higher)	⨁⨁◯◯ Low[a,c]	CRITICAL
**30s-sst**												
7	randomised trials	serious[a]	serious[b]	not serious	not serious	none	453	464	-	MD **2.68 higher** (0.86 higher to 4.49 higher)	⨁⨁◯◯ Low[a,b]	CRITICAL
**FES-I**												
3	randomised trials	serious[a]	not serious	not serious	serious[c]	none	192	149	-	MD **1.74 higher** (0.33 higher to 3.15 higher)	⨁⨁◯◯ Low[a,c]	CRITICAL
**6MWT**												
3	randomised trials	serious[a]	not serious	not serious	serious[c]	none	91	87	-	MD **22.54 higher** (4.56 lower to 49.64 higher)	⨁⨁◯◯ Low[a,c]	CRITICAL

a. Some studies had a high risk of bias or raised some concerns across various domains in the Cochrane RoB assessment

b. Substantial unexplained heterogeneity (I2 > 50%)

c. Limited sample size
